# CARF-dependent preferential RNA cleavage by Csm6 increases drug susceptibility of mycobacteria

**DOI:** 10.1093/nar/gkaf622

**Published:** 2025-07-02

**Authors:** Wenping Wei, Chun-Hui Gao, Xiaofang Jiang, Junjie Qiao, Li Zhang, Yunjun Yan, Guowei Zhao, Kaixin Yang, Jinyong Yan, Min Yang

**Affiliations:** Key Laboratory of Molecular Biophysics of the Ministry of Education, College of Life Science and Technology, Huazhong University of Science and Technology, Wuhan 430074, China; National Key Laboratory of Agricultural Microbiology, College of Resources and Environment, Huazhong Agricultural University, Wuhan 430070, China; National Key Laboratory of Agricultural Microbiology, College of Life Science and Technology, Huazhong Agricultural University, Wuhan 430070, China; National Key Laboratory of Agricultural Microbiology, College of Life Science and Technology, Huazhong Agricultural University, Wuhan 430070, China; National Key Laboratory of Agricultural Microbiology, College of Life Science and Technology, Huazhong Agricultural University, Wuhan 430070, China; Key Laboratory of Molecular Biophysics of the Ministry of Education, College of Life Science and Technology, Huazhong University of Science and Technology, Wuhan 430074, China; Key Laboratory of Molecular Biophysics of the Ministry of Education, College of Life Science and Technology, Huazhong University of Science and Technology, Wuhan 430074, China; Key Laboratory of Molecular Biophysics of the Ministry of Education, College of Life Science and Technology, Huazhong University of Science and Technology, Wuhan 430074, China; Key Laboratory of Molecular Biophysics of the Ministry of Education, College of Life Science and Technology, Huazhong University of Science and Technology, Wuhan 430074, China; Key Laboratory of Molecular Biophysics of the Ministry of Education, College of Life Science and Technology, Huazhong University of Science and Technology, Wuhan 430074, China

## Abstract

CRISPR-Cas systems are prokaryotic adaptive immune systems that defend against invading mobile genetic elements. The type III-A CRISPR-Cas system has been studied in the evolutionary and epidemiological context of *Mycobacterium tuberculosis*, the causative agent of tuberculosis. However, its biological function remains poorly understood. Here, we demonstrate that heterologous expression of *csm6*, a single-stranded RNA ribonuclease of the CRISPR-Cas system, exhibits preferential RNA cleavage activity targeting host transcripts. This activity significantly downregulates ribosomal and mycolic acid biosynthesis pathway genes, leading to a global reduction in translation levels and an increased drug susceptibility of *Mycobacterium smegmatis*. Furthermore, mutagenesis analysis revealed that Csm6’s biological function critically depends on its CARF domain rather than its HEPN domain. In conclusion, our study elucidates the biological role of the Csm6 protein in the CRISPR-Cas system, both *in vitro* and *in vivo*, highlighting how preferential RNA cleavage impacts multiple mycobacterial processes. These findings provide novel insights into the functional diversity of CRISPR-Cas systems in mycobacteria.

## Graphical Abstract

**Graphical Abstract:**
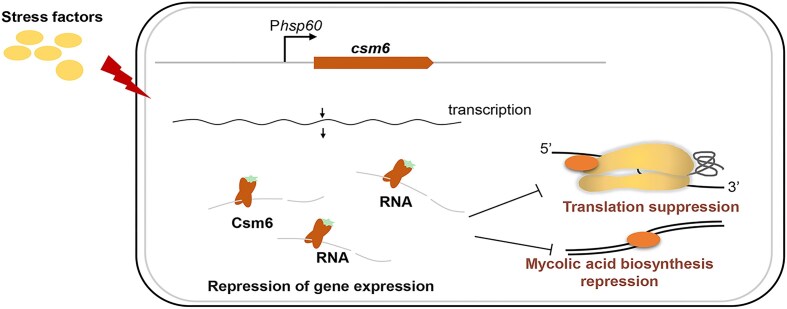

## Introduction

CRISPR-Cas systems are adaptive immune system in bacteria and archaea. They have 2 classes, 6 types, and 33 subtypes [1]. Among them, the type III-A CRISPR-Cas system is a distinctive feature of the *Mycobacterium tuberculosis* complex (MTBC), a group of organisms responsible for significant public health concerns [2]. Comparative analysis of 141 mycobacterial genomes revealed that the CRISPR-Cas system is exclusively present in MTBC, underscoring its unique association with the pathogenicity [3]. For instance, the CRISPR-Cas system of *M. tuberculosis* plays a crucial role in regulating bacterial virulence, gene expression, and growth under stress conditions [4, 5]. Exposure to capreomycin significantly increases the expression of *csm1*-*csm5* and *cas6* in the *M. tuberculosis* H_37_Rv strain, suggesting that CRISPR-associated genes are involved in drug resistance [6]. Furthermore, these *cas* genes are upregulated in the less virulent *Mycobacterium bovis* BCG strain when exposed to H_2_O_2_, and deletion mutants of the CRISPR loci exhibit increased sensitivity to H_2_O_2_, and reduced cell envelope integrity [4]. These studies collectively indicate that the CRISPR-Cas system in *M. tuberculosis* is closely associated with various pathogenic phenotypes, yet the nature of this relationship remains far from clear.

The type III-A CRISPR-Cas system is mediated by multi-subunit effector complexes Csm [7, 8]. In the complexes, the ribonuclease activity of Csm6 is required for anti-plasmid immunity [9]. Csm6 contain two highly conserved domains: the HEPN (Higher Eukaryotes and Prokaryotes Nucleotide-binding) domain and the CARF (CRISPR-associated Rossmann-fold) domain [10]. The ribonuclease activity of Csm6 is driven by the R-X_4-6_-H motif within the HEPN domain [9, 11], and is activated by a second messenger produced by the Type III interference complex, which consists of Csm1 to Csm5 [12]. Specifically, upon target RNA binding by the interference complex, the Cas10 (Csm1) subunit converts ATP into a cyclic oligoadenylate (cOA) product, which allosterically activates Csm6 by binding to its CARF domain, thus activate its nonspecific RNA degradation [13, 14]. In *M. tuberculosis*, CRISPR-Csm complexes structure represents the largest Csm complex reported thus far [15]. The bacterium achieves immunity against mobile genetic elements through a cyclic hexa-adenylate (cA6) signaling pathway [16, 17]. Therefore, the interplay between cA6 and Csm6 is essential for the bioactivity of the type III-A CRISPR-Cas system in *M. tuberculosis*.

Despite the ubiquitous distribution of the CRISPR-Cas system in the MTBC, *Mycobacterium smegmatis*, a commonly used model organism for studying mycobacteria, lacks this system in its genome [18]. *M. smegmatis* is a nonpathogenic species that grows more rapidly than other *Mycobacterium* species. However, its cell wall and lipid metabolism genes closely resemble those of *M. tuberculosis*, and it shares similar physiological and genetic characteristics [19, 20]. Since *M. smegmatis* lacks a native CRISPR-Cas system, introducing the type III-A CRISPR-Cas system from *M. tuberculosis* into *M. smegmatis* offers valuable insights into the biochemical properties of Cas proteins and their roles *in vivo*.

In this study, we first investigated the biochemical properties of Csm6, and then constitutively expressed *csm6* and its mutants in *M. smegmatis*, aiming to study the biochemical activity of Csm6 in mycobacteria. The results showed that Csm6 functions as a single-stranded RNA (ssRNA) ribonuclease independent of divalent metal ions. Transcriptomic analysis indicate that Csm6 affects the expression of numerous genes, particularly genes related to ribosome and mycolic acid biosynthesis pathways. Moreover, we proved the CARF domain of Csm6 is crucial for its activity. These insights contribute to a better understanding of the functional role of the CRISPR-Cas system and the mechanisms underlying bacterial stress responses.

## Materials and methods

### Bacterial strains and growth conditions

*Escherichia coli* DH5α and BL21 (DE3) strains (purchased from Novagen) and the corresponding transformed strains were cultivated in Luria–Bertani (LB) medium. *M. smegmatis* and *M. bovis* BCG were incubated at 37°C in Middlebrook 7H9 (BD, USA) broth supplemented with 0.2% glycerol, 0.025% Tween-80, or in 7H10 agar supplemented with 0.5% glycerol. When appropriate, kanamycin (50 μg/ml) and ampicillin (100 μg/ml) were added into the media. To obtain the overexpressing strain of *csm6*, DNA sequence encoding Csm6 was amplified from *M. tuberculosis (Mtb)* H37Ra genomic DNA by polymerase chain reaction (PCR), and cloned into a modified pMV261 vector. Subsequently, the resultant recombinant plasmid was transformed into wild-type *M. smegmatis*. Liquid culture and agar plate culture were performed at 37°C in a shaker and in incubator, respectively.

### Plasmid construction

All the primers, plasmids, and strains used in this study were listed in Supplementary Tables S1 and S2. To construct the constitutive expression plasmids, the open reading frame sequences of the CRISPR-Cas system genes (*cas2*, *cas1*, *csm6*-*csm1*, and *cas6*: *Rv2816c-Rv2824c*) were separately amplified using the genomic DNA of *Mtb* H37Ra (Accession number: ASM1614v1) as a template, and then cloned into the linearized pMV261 vector. Full-length *csm6* was also inserted into the pET28a-SUMO vector to obtain the N-terminal His6-fusion region plasmid. All plasmids were constructed using the HieffClone One Step Cloning Kit (YEASEN, 10 911, China). The point mutants of Csm6 (R324A, N325A, H329A) and the mutant dCsm6^CARF^ (S73A-G74A-T75A-P76A) were generated using the Mut Express II Fast Mutagenesis Kit (Vazyme, C214-01, China). The mutagenesis was performed following the manufacturer’s protocol, and the resulting mutants were confirmed by DNA sequencing.

### Protein expression and purification

N-terminal His6-fusion Csm6 recombinant plasmid and variants were transformed into the host strain BL21 (DE3) grown in LB medium. An overnight bacterium liquid was inoculated into 1 L fresh medium at 1% seed volume, and cultured at 37°C up to OD_600_ of 0.6. Proteins expression was induced by the addition of 0.5 mM isopropyl β-D-1-thiogalactopyranoside (CAS 367–93-1, Amresco, USA). The culture was continued for additional 16 h (hours) at 16°C. Cells were collected by centrifugation. The harvested cells were resuspended in Binding buffer (pH 8.0) containing 100 mM Tris–HCl, 500 mM NaCl, and 10 mM imidazole, and broken through sonication (300 W, 15 min). The lysates were centrifuged at 9000 × *g* for 30 min. The supernatants were loaded onto the affinity column (GE Healthcare, Germany), and washed with washing buffer (pH 8.0) containing 100 mM Tris–HCl, 500 mM NaCl, and 50 mM imidazole. The remaining proteins were then eluted using an elution buffer (pH 8.0) containing 100 mM Tris–HCl, 500 mM NaCl, and 250 mM imidazole. The purified proteins were dialyzed in the buffer containing 100 mM Tris–HCl, 10% glycerol for 2 h, and stored at −80°C. Protein concentrations were detected by Coomassie Brilliant Blue assay.

### Csm6 ribonuclease assay

All RNA and DNA oligonucleotides were purchased from TsingKe Company (Wuhan, China). To determine the ribonuclease activity of Csm6, gel-based electrophoresis assays were performed, as described previously [21]. Briefly, 1 μM Csm6 was incubated with 50 nM 3′-Cy5-labeled RNA (RNA1, 5′-ACUGCAACGCAAUAUACCAUAGCU-3′) in a 20 μl solution containing 20 mM 4-(2-hydroxyethyl)-1-piperazineethanesulfonic acid (HEPES) and 50 mM KCl (pH 7.5) at 37°C for 1 h. To determine Csm6 substrate specificity, double-stranded RNA (dsRNA) and double-stranded DNA (dsDNA) were prepared by annealing. Fluorescently labeled RNA1 and unlabeled RNA2 (the reverse complementary sequence of RNA1) were mixed at a ratio of 1:2, while fluorescently labeled DNA1 (the DNA equivalent of the RNA1 sequence) and unlabeled DNA2 were mixed at the same ratio. The obtained mixtures were heated to 65°C for 2 min in a water bath, then slowly cooled down to room temperature. The melting temperature (Tm) of all sequences was 54°C (Supplementary Table S2). The single-stranded DNA (ssDNA), dsDNA, dsRNA cleavage reaction systems were the same with ssRNA reaction system. To determine the effects of divalent metal ions on the cleavage activity, nuclease activity analysis was performed as follows. Mix different concentrations of ethylenediaminetetraacetic acid (EDTA) with 200 nM of RNA1 and 1 μM of Csm6 in a 20 μl reaction mixture containing 20 mM HEPES and 50 mM KCl (pH 7.5), and incubate at 37°C for 1 h. All reactions were terminated by adding RNA loading buffer (95% formamide, 5% glycerol, 0.005% bromophenol blue), followed by denaturation at 65°C for 5 min. The 20 μl of sample was loaded onto 20% polyacrylamide gel (8 M urea), and resolved in 0.5 × Tris-Borate-EDTA (TBE) buffer at 18 W for 1 h. The sample was visualized by a fluorescent displayer (GE Healthcare, Little Chalfont, Buckinghamshire, UK).

To evaluate the effect of cyclic adenylate on the enzymatic activity of Csm6, 0.25 μM of Csm6, dCsm6^CARF^, and dCsm6^CARF^ (H329A) were incubated with 200 nM of RNA1 in a total reaction volume of 20 μl, containing 20 mM HEPES and 50 mM KCl (pH 7.5). The reaction was initiated at 37°C by adding either cyclic hexa-adenylate (cA6, CAS 232933-63-0, BIOLOG Life Science Institute, Germany) or an equivalent volume of water. After a 30-min incubation, the reaction was terminated by adding an equal volume of RNA loading buffer. The products were then analyzed on a denaturing 20% polyacrylamide gel containing 8 M urea.

### Transcriptomic analysis

Transcriptomic analysis of each sample was performed in triplicate. All *M. smegmatis* wild-type strains, *csm6* overexpression, and dCsm6^CARF^ strains were cultured to an OD_600_ of 1.0 in 7H9 medium. The cells were harvested and then stored at −80°C until use. The extraction, library construction, and sequencing of total RNA were performed by Shanghai Majorbio Corporation (Shanghai, China). To compare the expression of different genes, mycobacterial genes with an adjusted *P*-value of < .05 and absolute log_2_ fold change of >2 were called as differentially expressed genes (DEGs). Gene Ontology (GO) enrichment analysis was conducted with R package ClusterProfiler. Raw RNA sequencing data has been deposited in the Sequence Read Archive (SRA), and are publicly accessible under the accession number PRJNA1218030 (https://www.ncbi.nlm.nih.gov/bioproject/PRJNA1218030).

### Quantitative real-time PCR assays

Quantitative real-time PCR (qRT-PCR) analysis was performed as described previously with some modifications [22]. Mycobacterial strains were grown to mid-log phase in their respective media at 37°C with shaking at 200 rpm. Cells from 30 ml cultures were harvested by centrifugation (4000 × *g*, 10 min, 4°C), and the pellets were immediately processed for RNA extraction. Total RNA was isolated from mycobacterial strains using TRIpure reagent (Aidlab, China) following the manufacturer’s protocol, with subsequent DNase I treatment (RNase-free, Thermo Scientific, USA) at 37°C for 30 min to eliminate genomic DNA contamination. Reverse transcription was performed using 5 × HiScript II qRT SuperMix II (Vazyme, China) with 1 μg RNA undergoing complementary DNA (cDNA) synthesis at 50°C for 15 min, followed by reaction termination at 85°C for 5 s in a Veriti thermal cycler. Gene-specific primer pairs (Supplementary Table S3) were used in real-time PCR analysis with 20 μl reactions containing 10 μl 2 × SYBR qPCR Master Mix (Vazyme, China), 1 μl cDNA, and 200 nM primers. The qRT-PCR was carried out using the ViiA™ 7 instrument (ABI Applied Biosystems, USA) under the following cycling parameters: 95°C for 5 min; 40 cycles of 95°C for 20 s, 60°C for 20 s, 72°C for 20 s; and melt curve analysis (95°C for 15 s, 60°C for 1 min, and 95°C for 15 s). The data were analyzed with QuantStudio Real-time PCR Software Version 1.3. The *sigA* gene encoding the primary sigma factor was used as the endogenous control based on its documented constitutive expression in mycobacterial growth phases [23, 24]. Gene expression levels were then calculated using the 2^−ΔΔCt^ method with normalization to *sigA* [25]. Two-tailed Student’s *t*-test was performed with error bars of 95% confidence intervals of three technical replicates.

### Polysome profiling

Polysome profiling was performed to analyze the distribution of ribosomes across different polysome fractions as previously described [26]. A 200 ml culture of pMV261 and *csm6*-expressing strains was grown to mid-log phase, filtered through 0.22-μm, 90-mm membranes (Millipore Sigma, USA) using a fritted glass microfiltration apparatus (Millipore Sigma, USA), and scraped into liquid nitrogen. For polysome extraction, the frozen samples were ground to a fine powder using RNase-free pestles and mixed with 750 μl of polysome extraction buffer (0.1 M KCl, 0.02 M HEPES-KOH [pH 7.4], 0.01 M magnesium acetate, 0.015 M 2-mercaptoethanol, 100 μg/ml cycloheximide). The mixture was vortexed thoroughly and incubated on ice for 5 min, followed by centrifugation at 15 000 × *g* for 10 min at 4°C. The resulting 300 μl supernatant was layered onto a 15%–50% linear sucrose gradient. The gradients were equilibrated at 4°C for 30 min. Subsequently, the sucrose gradients were centrifuged in a Beckman ultracentrifuge at 40 000 × *g* for 3 h at 4°C. The gradients were fractionated and analyzed using a gradient fractionator (BioComp Instruments, Canada). The polysome/monosome ratio was calculated based on the area under the curve (AUC) obtained from polysome profiling. The AUC for both the polysome and monosome fractions was quantified by measuring the intensity of the peaks corresponding to each fraction, and the polysome/monosome ratio was determined by dividing the AUC of the polysome fraction by that of the monosome fraction, providing a relative comparison of translation efficiency between the two strains.

### Membrane permeability experiment

Cultures of the *csm6*-expressing strain and the pMV261 control strain were grown to mid-log phase (OD_600_ = 0.6) in 7H9 medium. Cells were treated with 1 μg/ml Nile Red (Shanghai Yuanye Bio-Technology Co., Ltd, Cat# 7385-67-3) or 5 μg/ml propidium iodide (PI; Yuanye, Cat# 25535-16-4) for 30 or 120 min at 37°C with shaking. Post-incubation, 1 ml aliquots were pelleted by centrifugation (12 000 × *g*, 5 min, 4°C). For Nile Red-stained samples, pellets were washed three times with phosphate-buffered saline (PBS; pH 7.4) to remove unbound dye and resuspended in 1 ml PBS. PI-stained samples were resuspended directly in PBS without washing to avoid leakage of intracellular PI. Fluorescence intensity was measured using a Tecan Infinite 200 Pro microplate reader (Tecan, Switzerland) with excitation/emission wavelengths set to 488 nm/530 nm for Nile Red and 535 nm/617 nm for PI. Data were normalized to cell density (OD_600_) and analyzed from three biological replicates.

### Determination of mycobacterial growth curves and stress assays

Growth of the wild-type mycobacterial and all recombinant strains was examined according to the method described previously with some modifications [27]. All the recombinant strains were cultured to log phase at 37°C in 7H9 medium, then strain liquid was diluted to an OD_600_ of 0.1 in 100 ml 7H9 medium with 5 μg/ml isoniazid (INH) or without drug. The samples were collected at various time points, with equal time interval, and OD_600_ of the samples was measured.

The susceptibilities of the bacterial strains to H_2_O_2_ (0.125–2 μg/ml), rifamycin (RIF; 0.125–2 μg/ml), streptomycin (STR; 0.0625–1 μg/ml), and ethambutol (EMB; 0.125–2 μg/ml) were determined using the drug gradient dilution method. The pMV261 (empty vector), pMV261-*csm6* (*csm6*-expressing), and pMV261-d*csm6*^CARF^ (d*csm6*^CARF^-expressing) strains were cultured in 5 ml of 7H9 broth supplemented with appropriate antibiotics for plasmid selection. Overnight bacterial cultures were diluted to an OD_600_ of 0.1, and 1 ml of each bacterial suspension was inoculated into Polyamide bottles containing a series of serially diluted drugs and H_2_O_2_ in 4 ml of 7H9 broth. The cultures were incubated at 37°C for 16 h with shaking, and bacterial growth was assessed by measuring OD_600_ using a spectrophotometer. This procedure was repeated for each strain, and the susceptibilities to H_2_O_2_ and antibiotics were compared across the different strains.

For the drug plate assay, all bacterial cultures were grown to log phase, adjusted to an OD_600_ of 1.0, serially diluted (10^−1^–10^−3^), and spotted onto 7H10 medium supplemented with 5 or 10 μg/ml INH. The plates were incubated at 37°C for 3 days, and the results were scored based on colony growth. All experiments were performed in triplicate, and representative images are shown.

*M. bovis* BCG strains were constructed by electroporating the pMV261-*csm6* and pMV261-d*csm6*^CARF^ plasmids into wild-type BCG cells, followed by selection and validation to generate strains stably expressing *Mtb*Csm6 or the *Mtb*dCsm6^CARF^ mutant. Transformants were selected on 7H10 agar plates supplemented with 50 μg/ml kanamycin and 10% Oleic Acid–Albumin–Dextrose–Catalase (OADC). Validated strains were cultured in 7H9 medium containing 0.2% glycerol, 10% OADC enrichment, and 0.05% Tween 80 at 37°C with continuous shaking at 200 r/min. For drug susceptibility assays, mid-log phase cultures (OD_600_ = 1.0) were diluted to OD_600_ = 0.05 in fresh 7H9 medium and aliquoted into 5 ml plastic aliquot tubes. Each tube was treated with 0–2.5 μg/ml INH or 0–5 μg/ml EMB using a twofold serial dilution gradient. After 5 d of incubation at 37°C with shaking, bacterial growth was quantified by measuring OD_600_ using a spectrophotometer (Tecan, Switzerland).

### Growth profiling of mycobacterial strains under environmental stress conditions

To evaluate the growth of recombinant strains under different stress conditions, the *cas2*, *cas1*, *csm1*-*csm6*, *cas6*, and pMV261 (empty vector) expressing strains were cultured to the mid-log phase. One milliliter of each bacterial culture was inoculated into 4 ml of fresh 7H9 medium under the following stress conditions: for acid stress, the pH of the medium was adjusted to 4.5; for nutrient stress, the medium contained 0.05% glycerol, one-quarter of the concentration in normal medium; for oxidative stress, the medium was supplemented with 0.75 mM H_2_O_2_; and for lysozyme stress, 5 μg/ml lysozyme was added. The cultures were incubated at 37°C with shaking for 12 h, and bacterial growth was monitored by measuring OD_600_.

## Results

### *Mtb* Csm6 is a bona fide ssRNA ribonuclease

**Figure 1.**
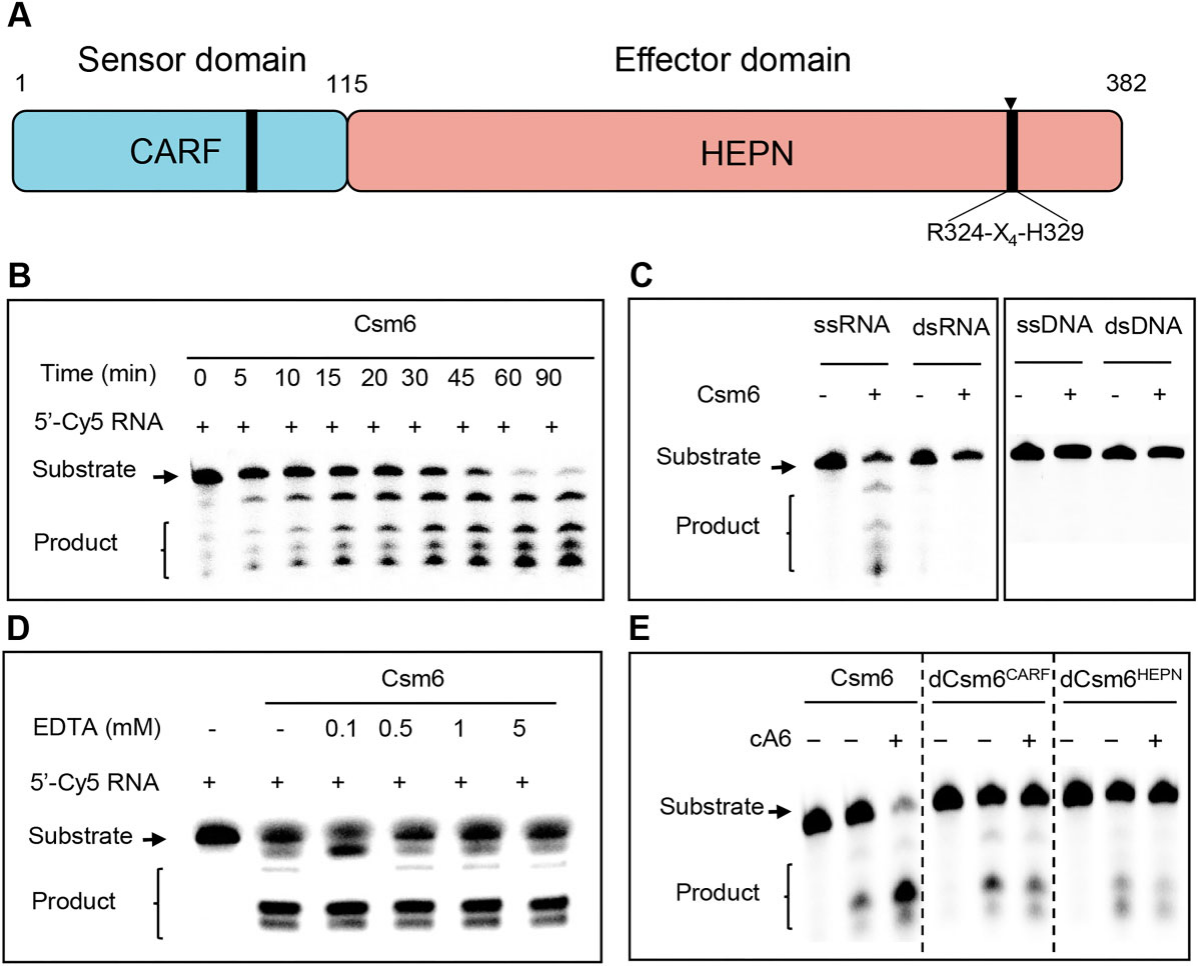
Csm6 is a metal ion-independent ssRNA ribonuclease. (**A**) Analysis of the domain structure of *M. tuberculosis* Csm6. Csm6 consists of 382 amino acids, of which the N-terminal has a CARF domain and the C-terminal has a HEPN domain. (**B**) Analysis of Csm6 nuclease activity. A 24 nt fluorescently labeled ssRNA (200 nM) was used as a substrate and incubated with Csm6 at 37°C for indicated time. (**C**) Specificity analysis of Csm6 nuclease activity. Different substrates (200 nM) were incubated with 1 μM Csm6 at 37°C for 1 h. (**D**) Analysis of the effect of divalent metal ions on Csm6 nuclease activity; 200 nM ssRNA substrate were incubated with 1 μM Csm6 in the presence EDTA at 37°C for 1 h. (**E**) Analysis of cA6-activated Csm6 nuclease activity; 0.25 μM wild-type Csm6, dCsm6^CARF^, and dCsm6^HEPN^ were incubated with 200 nM ssRNA with or without cA6 for 30 min. The results shown in the figure are representative results of two replicates of this experiment.

The Csm6 protein from *M. tuberculosis* contains an N-terminal CARF sensor domain and a C-terminal HEPN effector domain (Fig. 1A and Supplementary Fig. S1). To systematically characterized the biochemical activity of *Mtb* Csm6, we expressed and purified recombinant Csm6 protein and tested with a 24 nt 3′-Cy5 fluorescent-labeled RNA oligonucleotide. The results showed that short oligonucleotide products were generated after 5 min of reaction between Csm6 and the substrate, and after 60 min of reaction, the RNA substrate was almost completely degraded (Fig. 1B). In contrast, no cleavage was observed for dsRNA, ssDNA, and dsDNA (Fig. 1C). Notably, the cleavage of ssRNA is not affected by increasing concentrations of EDTA, indicating that the activity of Csm6 is independent of metal ions (Fig. 1D).

The multiple sequence alignment of Csm6 amino acid sequences from *Thermus thermophilus*, *Staphylococcus epidermidis*, *Lactobacillus acidipiscis*, *M. bovis* BCG, and *M. tuberculosis* revealed a low sequence identity between species (9% and 28%). However, key motifs in the CARF and HEPN domains, including SGTP and R-X_4-6_-H, were highly conserved (Supplementary Fig. S2). SGTP has been identified as the ligand-binding interface of the CARF domain, while R-X_4-6_-H serves as the substrate-binding site of the nuclease [13, 21]. To investigate these regions, we replaced Ser73-Pro76 with Ala to create the dCsm6^CARF^ variant and introduced H329A mutation. Subsequently, we assessed the ligand-binding functionality of both the CARF and HEPN domains. Indeed, the presence of cA6 significantly enhances the nuclease activity of Csm6 compared to conditions without cA6 (Fig. 1E, lanes 1–3). In contrast to wild-type Csm6, the dCsm6^CARF^ mutant no longer responded to cA6 (Fig. 1E, lanes 4–6). Additionally, the HEPN potential active site mutant (H329A) exhibits a significant loss of nuclease activity, as demonstrated by the absence of significant short oligonucleotide products in both the presence and absence of cA6 (Fig. 1E, lanes 7–9). These results confirm that *Mtb* Csm6 is a metal ion-independent ssRNA ribonuclease, and that its core motif binds to the second messenger cA6 *in vitro*, exhibiting high conservation across orthologs from other bacterial species.

### Csm6 exhibits preferential RNA cleavage activity to host transcripts

**Figure 2.**
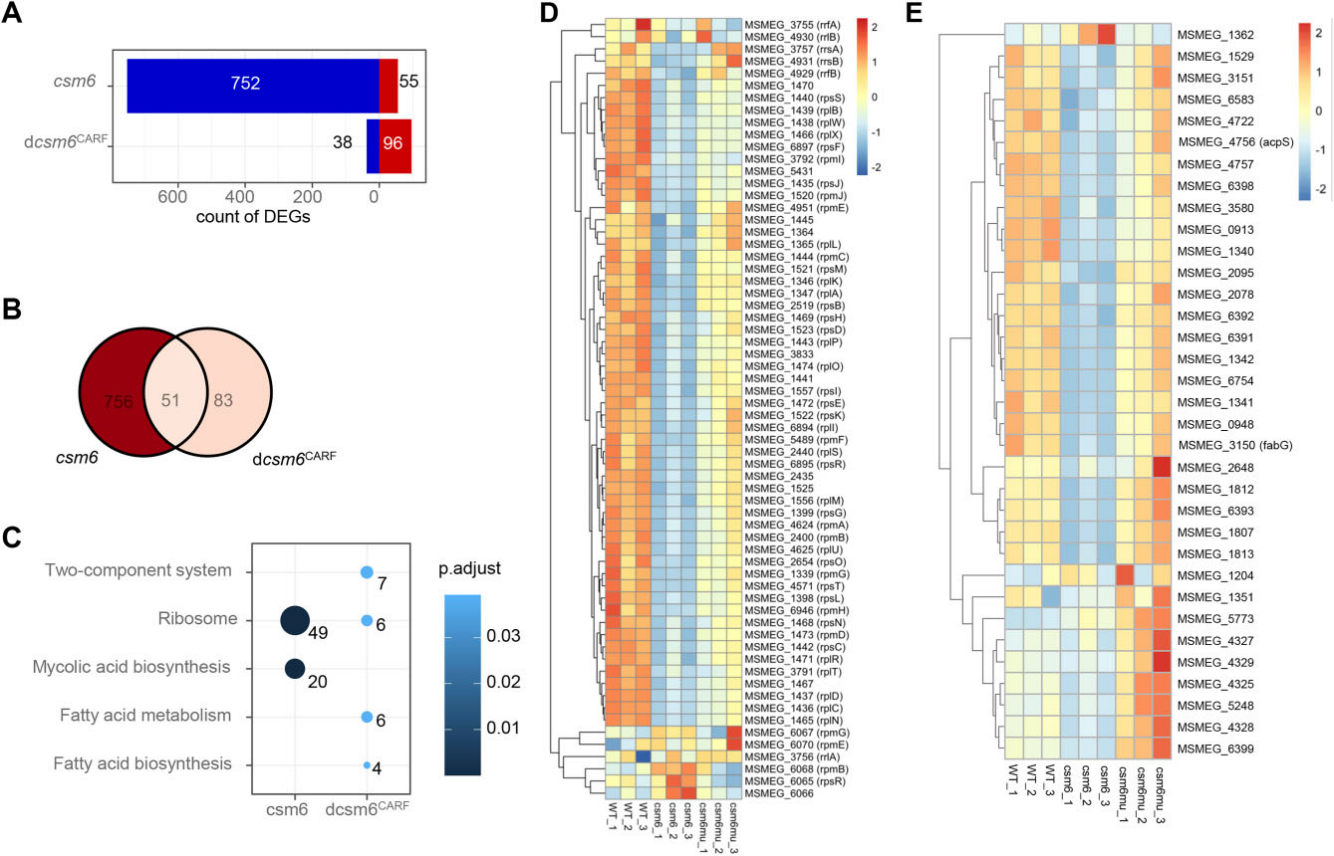
Transcriptome analysis of the effect of Csm6 on gene expressions of *M. smegmatis*. (**A**) Number of DEGs in *csm6*-expressing and d*csm6*^CARF^-expressing mutant strains. Blue, downregulated. Red, upregulated. (**B**) Overlap of DEGs. (**C**) KEGG enrichment analysis of DEGs. Over-representation test was performed with clusterProfiler, and the results were visualized with dotplot. Number of genes were labeled on the side. Heatmap showing the normalized expression levels of ribosomal protein genes (**D**) and mycolic acid biosynthesis genes (**E**) under different experimental conditions. Rows represent individual genes, clustered based on their expression profiles, while columns represent distinct experimental conditions, ordered as provided. The color scale indicates gene expression levels.

Given that Csm6 is a nonspecific nuclease that cleaves ssRNA, it cannot distinguish between host and phage RNA, therefore, it may also degrade the bacterial self messenger RNA (mRNA) and thus lower gene expressions *in vivo*. To test this hypothesis, we introduced the wild-type *csm6* and d*csm6*^CARF^ mutant genes into *M. smegmatis* to obtain constitutively expressed strains. Subsequently, gene expression in the recombinant strains was assessed using RNA-seq. A total of 807 DEGs were detected in the *csm6*-expressing strain, with 55 genes upregulated and 752 genes downregulated (|log2FC| > 0.5, p. adjust < .05, same below). Conversely, in the d*csm6*^CARF^-expressing strain, only 96 genes were upregulated while 38 genes were downregulated (Fig. 2A). Except for 51 overlapping DEGs, most were specific to the presence of the wild-type Csm6 protein rather than dCsm6^CARF^ (Fig. 2B). Although numerous DEGs were found in the *csm6*-expressing strain, Kyoto Encyclopedia of Genes and Genomes (KEGG) pathway enrichment analysis revealed that only two pathways, which are ribosome (p. adjust = 1.1E-24), and mycolic acid biosynthesis (p. adjust = 1.5E-6), were significantly enriched in these DEGs (Fig. 2C–E and Supplementary Fig. S3). Specifically, 49 out of all the ribosome pathway 64 genes annotated have significantly downregulated (Supplementary Fig. S4). By contrast, although ribosome pathway was also enriched in d*csm6*^CARF^-expressing strain, the count of genes was decreased from 49 to 6 and the significance level was much higher (p. adjust = 0.039) (Fig. 2C).

Ribosome pathway genes are mainly large subunit and small subunit structural genes, and almost all of them were found downregulated in *csm6*-expressing strain, and recovered in the d*csm6*^CARF^-expressing strain (Fig. 2D). To validate the transcriptome data, qRT-PCR assays were performed on both *csm6-* and d*csm6*^CARF^-expressing strains. Consistent with the RNA-seq data, qRT-PCR analysis confirmed decreased expression of multiple ribosomal protein genes, including those encoding the small subunits of ribosome (*rpsJ* and *rpsT*), as well as the large subunits of ribosome (*rplC*, *rplN*, *rpmF*, and *rpmG*), while the expression of internal control *sigA* remained stable (Supplementary Fig. S5A). Intriguingly, the d*csm6*^CARF^-expressing strain exhibited a reciprocal upregulation of these genes (Supplementary Fig. S5B), suggesting that CARF deletion reverses Csm6-mediated transcriptional repression of ribosomal pathways.

**Figure 3.**
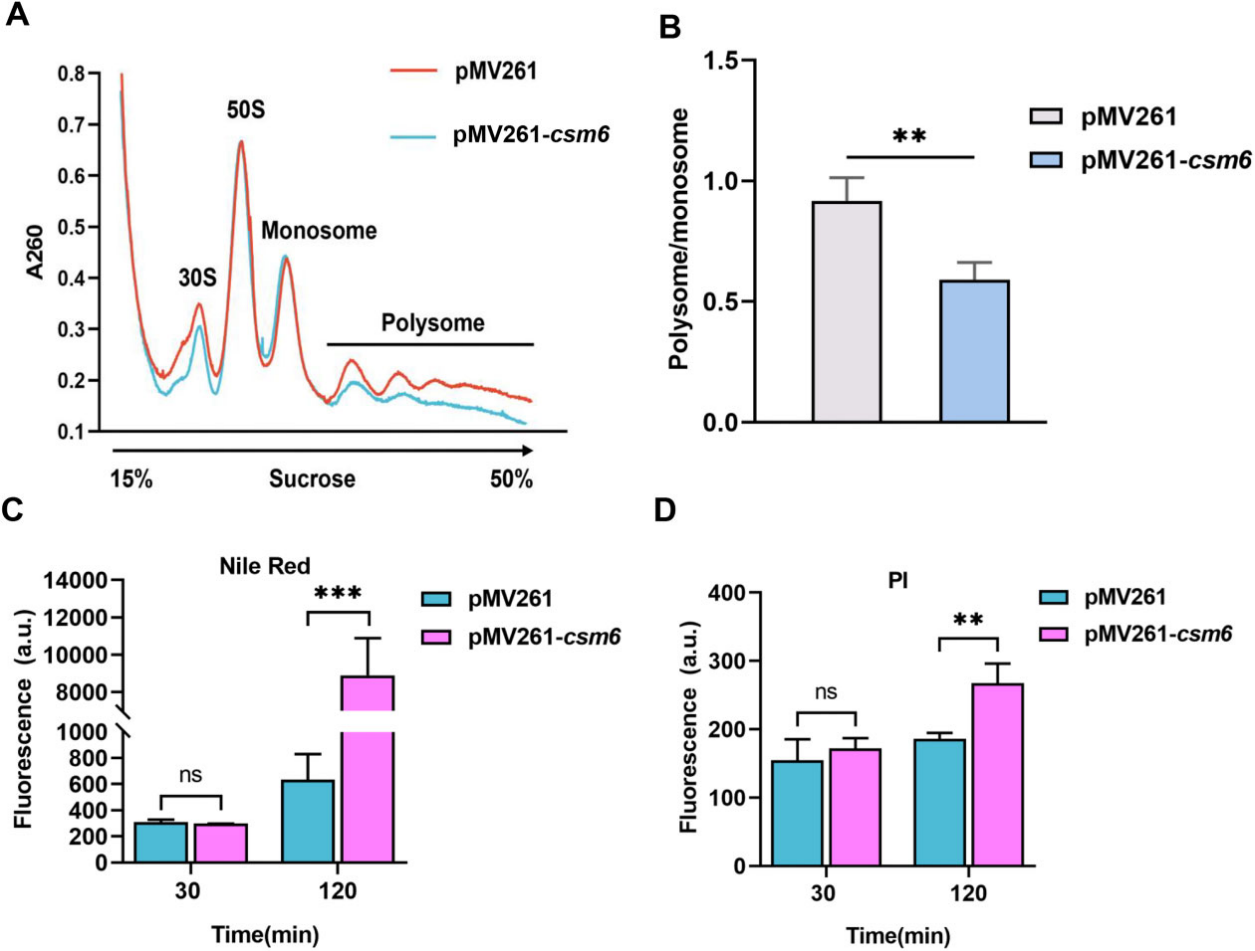
Csm6 inhibits overall translation and weakens cell wall integrity. Ribosome profile analysis of the distribution of polysome and monosome in pMV261 and *csm6*-expressing strains (**A**) and statistics of the ratio of polysome to monosome content in the two strains (**B**). This ratio was calculated using the AUC derived from polysome profiling, with the AUC values for polysome and monosome fractions quantified from their respective peaks in the polysome profiles. (**C**) PI uptake assay. Fluorescence intensity (ex/em 535/617 nm) was measured at 30 and 120 min post-treatment. (**D**) Nile red staining assay. Fluorescence intensity (ex/em 488/530 nm) was measured at 30 and 120 min post-treatment. *P*-values were calculated by GraphPad Prism 8 with independent samples two-tailed Student’s *t*-test. The asterisks represent significant differences between the two groups (*P < .05, **P < .01, ***P< .001).

Previous studies have shown that reduced ribosomal protein gene expression impacts global translation efficiency [28, 29]. To investigate whether Csm6 impairs the global translation efficiency, we conducted polysome profiling experiments to compare the translation characteristics of wild-type and the *csm6*-expressing strains using sucrose gradient centrifugation (Fig. 3A). By calculating the translation efficiency of WT and *csm6* strains, we found that the polysome/monosome ratio of the *csm6*-expressing strain was approximately half that of the WT strain, indicating a lower overall translation level in the *csm6*-expressing strain (Fig. 3B). This finding demonstrating that presence of Csm6 inhibits the overall cytoplasmic translation in mycobacteria.

Aside from the enrichment of ribosome pathway in *csm6*-expressing strain, the mycolic acid biosynthesis pathway is also enriched (Fig. 2C and E), suggesting that Csm6 activity may compromise cell wall integrity. To validate this hypothesis, we first performed qRT-PCR analysis, which subsequently confirmed significant downregulation of key mycolic acid biosynthesis genes (Supplementary Fig. S5C and D). Next, we assessed membrane integrity by treating log-phase cultures with the membrane-permeant fluorescent dye PI and the hydrophobic fluorescent dye Nile red. While fluorescence intensity for both dyes in the *csm6*-expressing strain remained comparable to controls at 30 min, it showed a significant increase after 120 min (Fig. 3C and D), indicating an increase in membrane permeability. Collectively, these results demonstrate that Csm6 expression suppresses the transcription of mycolic acid biosynthesis genes, thereby impairing cell wall integrity.

Taken together, these results demonstrate that Csm6 actively cleaves RNA, specifically suppressing ribosome translation and impairing cell wall integrity. This suggests that Csm6 preferentially cleaves host RNAs *in vivo*.

### Csm6 modulates drug susceptibility and stress response

Mycolic acids are long-chain, branched fatty acids that are key components of the mycobacterial cell wall, and they are essential for the pathogen’s survival, virulence, and resistance to environmental stressors, including antibiotics [30, 31]. Given that anti-tuberculosis drugs primarily target cell wall synthesis (e.g. INH, EMB), RNA transcription (RIF), and ribosomal translation (STR) [22, 27], we subsequently investigated the role of Csm6 in regulating bacterial growth under treatment with these antibiotics.

**Figure 4.**
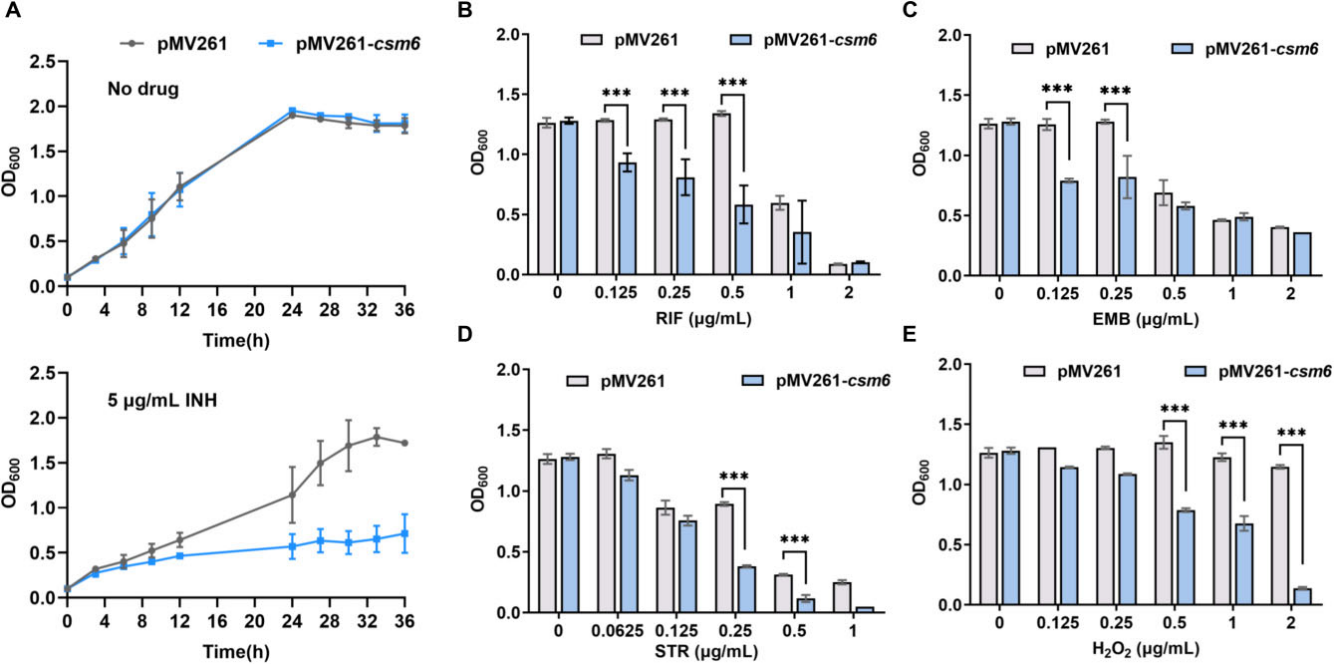
Csm6 mediates susceptibility of *M. smegmatis* to isoniazid. (**A**) Growth curve of the effect of Csm6 on bacterial INH susceptibility. The *csm6*-expressing strain and control strain were grown in 7H9 media with no drug (left panel) or 5 μg/ml INH (right panel). The recombinant strains were grown in 7H9 medium supplemented with different concentrations of drugs: (**B**) rifampicin, (**C**) EMB, (**D**) STR, (**E**) H_2_O_2_. The results in figure represent the average and standard deviation of three independent biological experiments. *P*-values were calculated by GraphPad Prism 8 with unpaired two-tailed Student’s *t*-test. Asterisk represents the significant difference between the data of each concentration and the data of no drug (*P < .05, **P < .01, ***P < .001).

First, we assessed the drug susceptibility of the *csm6*-expressing strain to INH. In the absence of INH, no significant difference in growth was observed between the *csm6*-expressing strain and the control strain (Fig. 4A). However, when cultured in medium containing 5 μg/ml INH, the growth of the *csm6*-expressing strain was significantly inhibited compared to the control strain (Fig. 4A), indicating increased susceptibility to the antibiotic. Meanwhile, we assessed the sensitivity of the *csm6*-expressing strain to RIF, EMB, and STR, using the drug gradient dilution method. After 16 h of drug treatment, the optical density of the *csm6*-expressing strain was notably lower than that of the pMV261 strain, particularly at concentrations such as 0.25 μg/ml and 0.5 μg/ml for RIF, 0.125 μg/ml and 0.25 μg/ml for EMB, and 0.125 μg/ml and 0.25 μg/ml for STR (Fig. 4B–D). Nutrient-limited growth assays (0.05% glycerol medium) demonstrate that the *csm6*-expressing strain remains sensitive to INH and EMB (Supplementary Fig. S6A–C), indicating that Csm6 expression does not cause general growth defects that would make cells more susceptible to drugs. Moreover, Csm6 expression does not trigger an SOS response (Supplementary Fig. S7A and B).

**Figure 5.**
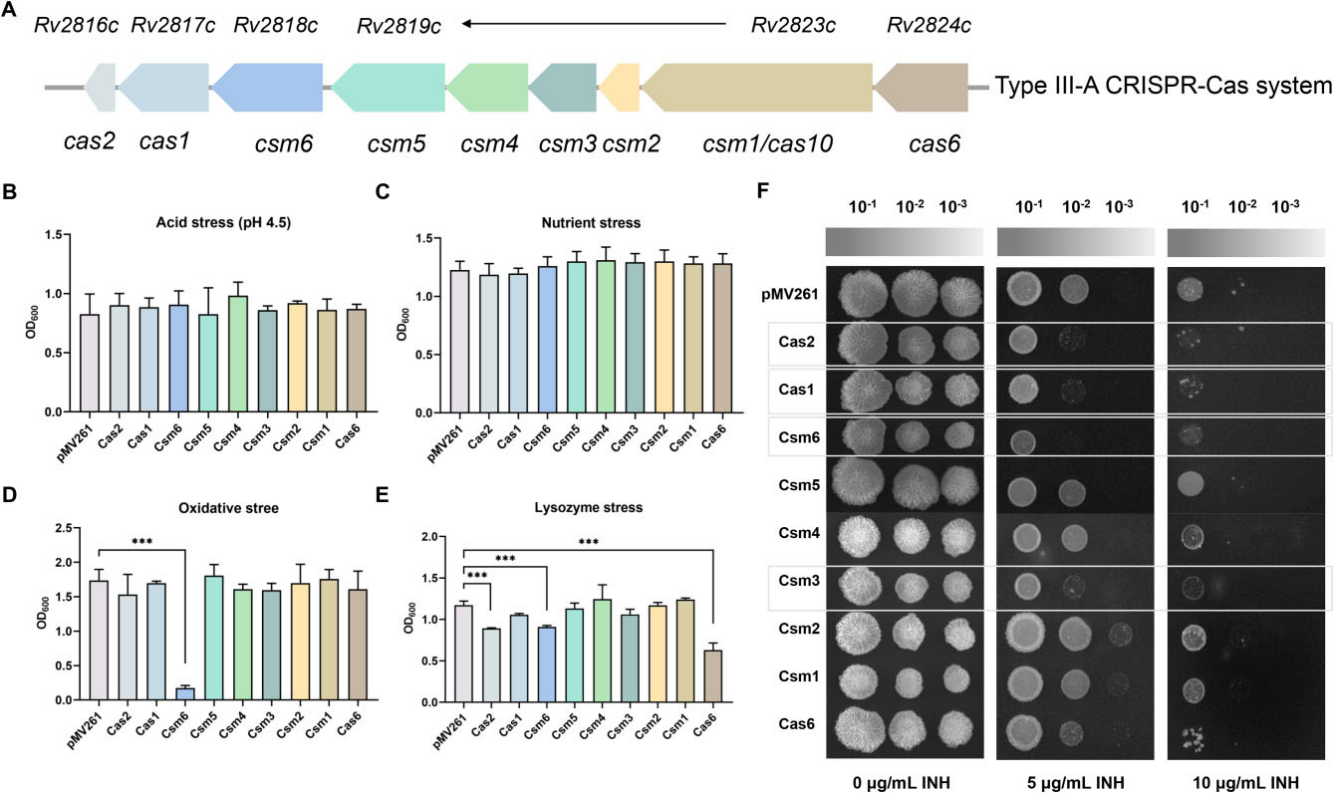
Growth of *cas* gene overexpression strains under environmental stress. (**A**) The CRISPR-Cas locus in *M. tuberculosis*. Growth of recombinant strain under acid stress (**B**), nutrient stress (**C**), oxidation stress (**D**), and lysozyme stress (**E**). The recombinant strains grew to mid-log phase, and each bacterial liquid was inoculated into 4 ml of fresh 7H9 medium and cultured for 12 h. The culture conditions were as follows: the pH of the acid stress medium was 4.5; the glycerol content of the nutrient stress medium was 0.05%, 1/4 of that of the normal medium; the oxidative stress medium contained 0.75 mM H_2_O_2_; the lysozyme stress medium contained 5 μg/ml Lysozyme. The abscissa represents the *cas* gene expressing strains. *P*-values were calculated by GraphPad Prism 8 with a two-tailed Student’s *t*-test. Asterisk represents a significant difference between two groups (***P < .001). (**F**) Growth of recombinant strains under INH stress. The *cas* gene overexpressing and control strains were grown in 7H9 medium to mid-log phase. The cultures were diluted to 10^−1^–10^−3^, and the same amount of bacterial liquid was spotted on 7H10 plates supplemented with 0 μg/ml INH, 5 μg/ml INH, and 10 μg/ml INH. Then, the plates grow for a further 3 day at 37°C.

Finally, we compared the roles of Csm6 and other Cas proteins in the CRISPR system in responding to various environmental stresses. Similarly, all *cas* genes were constitutively expressed in *M. smegmatis* (Fig. 5A). The study evaluated the impact of Cas proteins on bacterial survival under various stress conditions, including acidic stress, nutritional stress, oxidative stress, lysozyme stress, and antibiotic stress. Under pH 4.5 and nutrient-limited medium conditions, although the overall bacterial concentration decreased significantly, the growth rate of the overexpression strains exhibited no significant differences compared to the pMV261 control strain (Fig. 5B and C). Notably, under 0.75 mM H_2_O_2_ treatment, the growth of the *csm6* strain was markedly inhibited compared with the control strain, whereas other strains did not exhibit this phenotype (Fig. 5D). Additionally, besides the compromised lysozyme resistance in the *csm6* strain, the expression of *cas2* (associated with phage spacer acquisition) and *cas6* (involved in CRISPR RNA (crRNA) processing maturation) also resulted in reduced bacterial survival (Fig. 5E). Furthermore, the strains of expressing *cas2*, *cas1*, and *csm3* were also more sensitive to INH (Fig. 5F).

These findings collectively indicate that the CRISPR-Cas system, especially the Csm6 protein, mediates mycobacterial responses to multiple stresses, including oxidative stress, lysozyme stress, and drug stress.

### The CARF domain is crucial for Csm6 biological function *in vivo*

**Figure 6.**
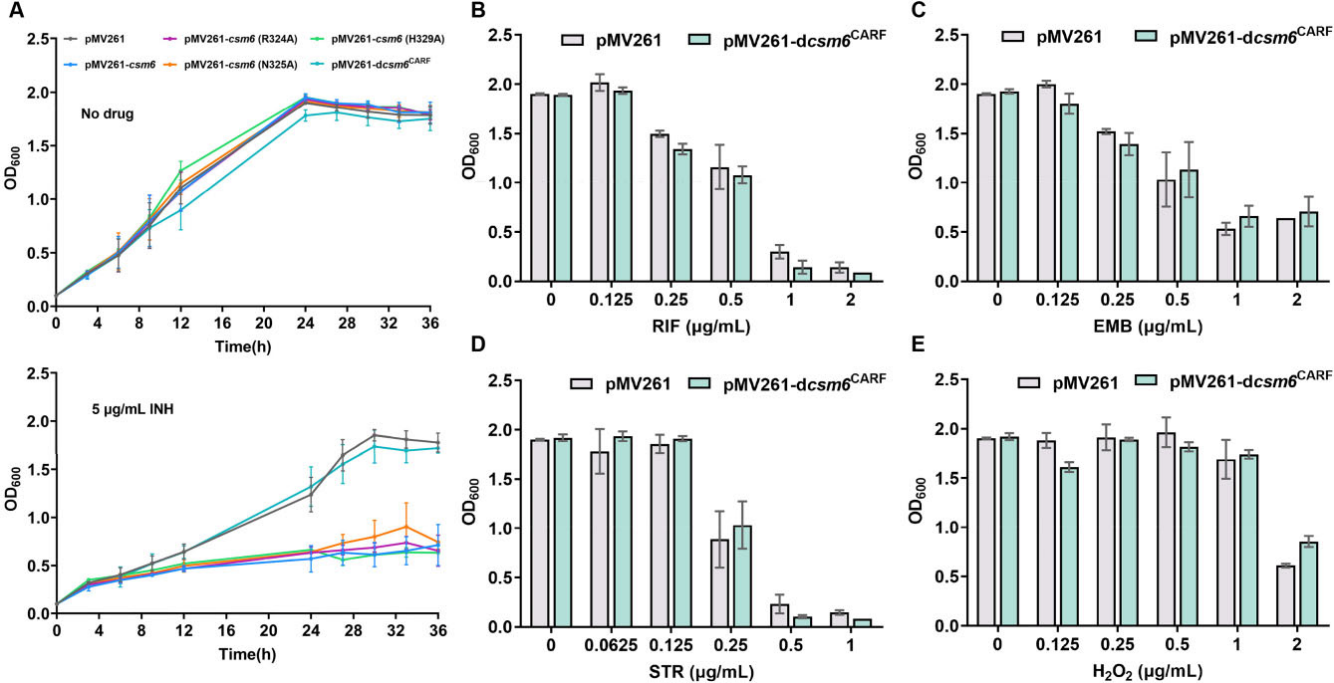
Effect of CARF as a key domain of Csm6 on ribonuclease activity and mycobacterial drug susceptibility. (**A**) Growth analysis of Csm6 HEPN domain and CARF mutants in the presence of INH. Growth curves of pMV261, pMV261-*csm6*, pMV261-*csm6* (R324A), pMV261-*csm6* (N325A), pMV261-*csm6* (H329A), and pMV261-d*csm6*^CARF^ in the absence (above) or presence (below) of 5 μg/ml INH in 7H9 medium. Growth analysis of d*csm6*^CARF^ mutant strain in the presence of multiple anti-tuberculosis drugs: (**B**) rifampicin, (**C**) EMB, (**D**) STR, (**E**) and H_2_O_2_. The results in figure represent the average and standard deviation of three independent biological experiments. *P*-values were calculated by GraphPad Prism 8 with unpaired two-tailed Student’s *t*-test. Asterisk represents the significant difference between the data of each concentration and the data of no drug (*P < .05, **P < .01, ***P < .001).

The biological function of Csm6 should come from either CARF or HEPN domains. First, we questioned whether the Csm6-mediated phenotype of Mycobacteria to drugs is related to its ribonuclease activity. To test this hypothesis, we constructed *M. smegmatis* strains with single point mutations at the active site of the Csm6 HEPN domain (R324A, N325A, and H329A) and evaluated their growth in the presence of INH. Unexpectedly, the growth of R324A, N325A, and H329A strains were still significantly inhibited by INH, similar to the *csm6*-expressing strain (Fig. 6A). These results emphasize that Csm6-mediated ribonuclease activity is not the primary cause of mycobacterial drug susceptibility.

The CARF domain binds cyclic adenosine monophosphate molecules, regulating the activity of downstream effector proteins. Consequently, the CARF sensor domain of Csm6 might sense nucleotide-derived signaling molecules to regulate gene transcription, thereby affecting the bacterium’s ability to respond to environmental stress. To test this, we examined the growth curves of Csm6 CARF domain mutants in the presence of INH. In the presence of INH, the growth of the d*csm6*^CARF^ mutant reverted to the same trend as the wild-type strain, eliminating INH susceptibility (Fig. 6A). We further measured the growth of the d*csm6*^CARF^ mutant under RIF, EMB, STR drug stress. The results showed that the growth of the d*csm6*^CARF^ mutant was essentially the same as the control strain (Fig. 6B–D). These results indicate that the mechanism by which Csm6 responds to drug and oxidative stress is mediated through its CARF domain. These findings clearly show that disrupting the CARF domain eliminates the Csm6-mediated susceptibility of mycobacteria to anti-TB drugs.

To assess the broader relevance of Csm6’s role in mycobacterial drug susceptibility, we engineered *csm6* and its CARF domain mutant in *M. bovis* BCG, a surrogate strain for *M. tuberculosis*. Phenotypic assays revealed that the *csm6*-expressing BCG strain exhibited enhanced susceptibility to INH and EMB compared to the pMV261 control strain, whereas the d*csm6*^CARF^ mutant showed no significant difference from the vector control (Supplementary Fig. S8A and B). Furthermore, qRT-PCR analysis revealed downregulation of mycolic acid biosynthesis genes (*fbpC*, *fbpD*, *fadD32*) in the *csm6*-expressing BCG strain (Supplementary Fig. S8C). All the results are consistent with the findings observed in *M. smegmatis* (Fig. 2C), thus demonstrate that the CARF domain is essential for Csm6-mediated modulation of mycobacterial stress tolerance.

## Discussion

Given the complex gene composition, and their widespread in archaea, the type III system CRISPR-Cas system appears to be the best candidate for the ancestral state [1]. The Csm6 protein, a component of the Type III-A CRISPR-Cas system, plays a crucial role in bacterial immune responses by nonspecifically degrading RNA from invading plasmids and viruses. Unlike other CRISPR-associated RNases, Csm6 operates independently of the Csm/Cmr complex, yet it possesses distinct ssRNA ribonuclease activity that does not require bivalent metal ions. Here, we provide biochemical evidence that *Mtb* Csm6 is a ssRNA ribonuclease independent of bivalent metal ions, capable of binding cA6 to nonspecifically cleave ssRNA (Fig. 1). This is consistent with the catalytic activity of other HEPN domain ribonucleases Csx1, Ire1, and RNase L [7, 11, 21]. Furthermore, we demonstrate that the SGTP motif within the CARF domain plays a crucial role in ligand binding, highlighting its importance in the protein’s functional activity (Fig. 1E and Supplementary Fig. S2). However, our results showed that the cleavage of Csm6 exhibits preferential to ribosomal and mycolic acid synthesis gene transcripts *in vivo* (Fig. 2C and Supplementary Fig. S4). Notably, a recent publication revealed that selective degradation of phage RNAs by the Csm6 ribonuclease provides robust type III CRISPR immunity in *Streptococcus thermophilus* [32]. Although these evidences could not turn over that Csm6 is nonspecific in degrading ssRNA, the preferences of cleavage should be a normal situation in its action.

The independent nuclease activity of Csm6 ensures effective immunity even when DNA targeting efficiency is compromised [8, 16]. Although effective, this response may cause temporary growth stasis [33, 34]. Csm6 activity can be regulated through cOA synthesis; for instance, cyclic nucleases terminate the antiviral state by generating linear adenosine from cyclic tetraadenylate [35]. In some bacteria, the CARF domain of Csm6 acts as both a cOA sensor and cyclic nuclease, allowing precise control over cOA degradation and RNase inactivation [36, 37]. In our study, we observed that in *M. smegmatis*, Csm6 overexpression, but not the CARF mutation, can render bacteria more vulnerable to drugs, suggesting a regulatory role of this domain in stress response (Figs 4 and 6). This provides strong evidence that the CARF domain mediates nuclease activation in Csm6, thereby influencing the bacteria’s response to environmental stress. Despite of this, our results indicate the regulation of Csm6 should be more complex than expected. The questions are: (i) given a host without CRISPR-Cas complex genes (especially *cas10*), whether the signaling molecule cA6 can still be generated? (ii) if not, is there any other molecule that can be used to trigger the biochemical activity of Csm6 *in vivo*?

To address the above-mentioned questions, we performed targeted metabolomic analysis of nucleotide-derived signaling molecules and confirmed that no cA6 was detectable in the *csm6*-expressing *M. smegmatis* strain, consistent with the absence of endogenous Cas10 required for cA6 synthesis in canonical Type III CRISPR-Cas systems (Supplementary Fig. S9A). However, targeted metabolomics revealed elevated levels of cyclic nucleotides cAMP and cGMP in the *csm6*-expressing strain (Supplementary Fig. S9B and C). To test whether these molecules could directly activate Csm6, we supplemented purified Csm6 with cAMP or cGMP (1 mM) *in vitro*. No enhancement of RNase activity was observed (Supplementary Fig. S9D), suggesting that cAMP/cGMP do not act as direct ligands for Csm6 activation. This discrepancy between *in vivo* metabolite accumulation and *in vitro* inactivity implies that Csm6’s physiological activation in mycobacteria may require additional cofactors (e.g. protein interactors) or post-translational modifications triggered by RNA cleavage-induced stress, rather than cyclic nucleotides alone. Additionally, an alternative activation mechanism may involve linear polyadenylated RNAs. In *Sulfolobus islandicus*, the CARF domain ribonuclease Csx1 is allosterically activated by RNAs carrying tetraadenylate (polyA4) tails through its N-terminal sensory domain [38]. Although Csm6 belongs to a distinct CRISPR subtype (III-A vs. III-B), its conserved CARF domain architecture suggests potential sensitivity to polyadenylated RNA species generated during stress-induced transcriptional reprogramming. Further studies, including proteomic profiling of Csm6-associated complexes and systematic screening of RNA ligands with varying 3′-tail configurations, are warranted to resolve this mechanism.

Recent studies indicate that the CRISPR-Cas system has evolved beyond its traditional role in adaptive immunity, becoming a vital mechanism for bacteria to regulate growth, gene expression and virulence [39–42]. To further investigate Csm6’s *in vivo* impact, we conducted stress response experiments exposing *M. smegmatis* to oxidative stress (H_2_O_2_) and lysozyme, in addition to antibiotic treatments. Overexpression of Csm6 diminished the bacterial resistance to these stresses, implying that Csm6’s nonspecific RNA degradation not only disrupts normal cellular processes but also heightens susceptibility to external stressors (Fig. 5). This phenotype is mechanistically attributed to CARF domain-dependent suppression by Csm6 of ribosomal protein genes and mycolic acid biosynthesis genes, as revealed by RNA-seq and qRT-PCR analyses (Fig. 2 and Supplementary Fig. S5). This dual transcriptional repression results in a global reduction in cellular translation rates and compromised cell wall integrity, synergistically sensitizing mycobacteria to environmental and pharmacological stresses. Additionally, we observed a significant increase in the expression of ribosomal genes in the d*csm6*^CARF^-expressing strain (Supplementary Fig. S5A). This is likely because the loss of the CARF domain disrupts Csm6’s RNA cleavage activity, relieving translational suppression and triggering compensatory upregulation of ribosomal genes to restore translational homeostasis. Furthermore, we observed that heterologous expression of the *Mtb csm6* in *M. bovis* BCG recapitulated the drug susceptibility phenotype and downregulation of mycolic acid biosynthesis genes (Supplementary Fig. S8A–C). It is noteworthy that the *csm6* gene in *M. bovis* BCG is naturally truncated compared to *Mtb csm6* [43]. These findings demonstrate that the function of Csm6 is universally conserved across mycobacterial species, including both pathogenic and nonpathogenic lineages. While prior studies have implicated the Type III-A CRISPR-Cas system in mycobacterial oxidative stress responses [4], our findings reveal a CARF-dependent mechanism by which Csm6 reshapes redox homeostasis (Fig. 5D). The selective nonresponsiveness of the CARF mutants to these stresses underscores the domain’s role in mediating stress response, as it effectively “senses” the environmental triggers that activate Csm6’s RNA degradation activity. This highlights the CARF domain’s importance in regulating cellular resilience under stress conditions, aligning with its known function in binding and regulating cOA degradation through ring nuclease activity [36, 37].

**Figure 7.**
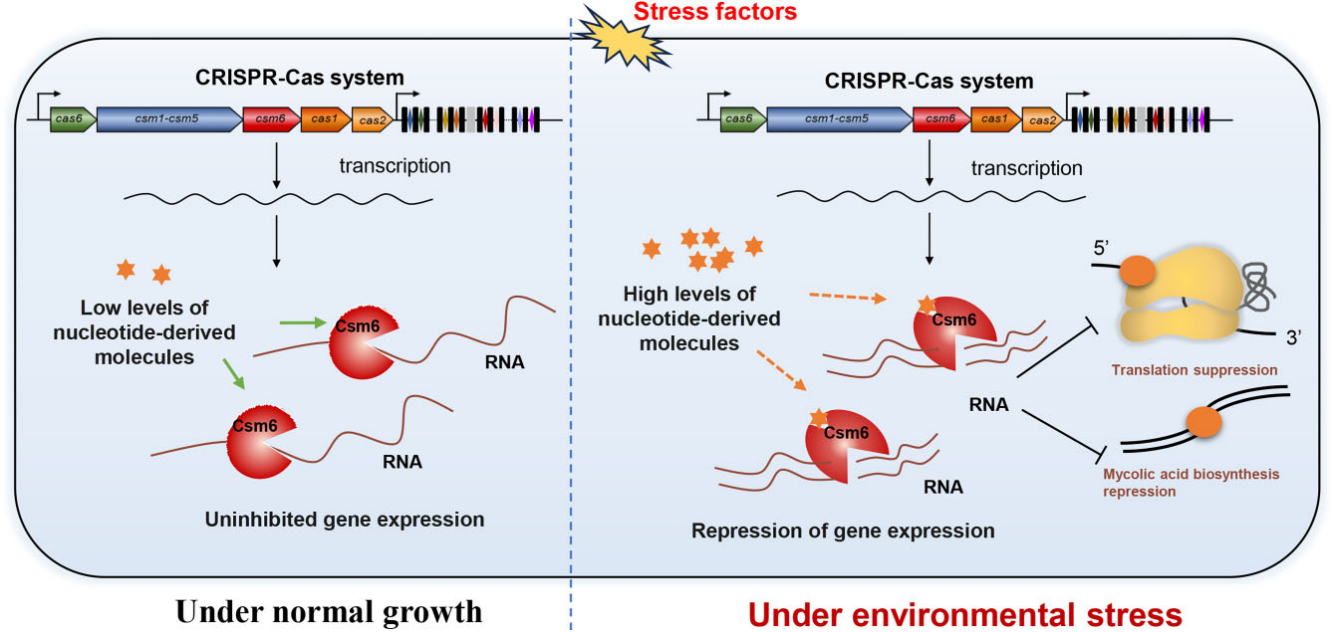
Schematic of the mycobacterial stress response mediated by Csm6 in the CRISPR-Cas system. Under normal conditions, bacteria synthesize low levels of nucleotide-derived molecules, which does not activate Csm6, allowing RNA transcription and bacterial growth to continue. However, under environmental stress, stress factors stimulate bacteria to produce elevated levels of nucleotide-derived molecules, which bind to Csm6 and activates its ssRNA ribonuclease activity. This activation induces degradation of mRNA from ribosomal protein genes and mycolic acid biosynthesis genes, disrupting protein translation and reducing membrane permeability. Consequently, bacterial metabolic homeostasis is compromised, rendering the cells more susceptible to antibiotic-induced stress.

Based on the above findings, we propose a hypothesis as follows: under normal growth conditions, bacteria generate low levels of nucleotide-like molecules, which do not activate Csm6. Consequently, RNA transcription proceeds unimpeded, supporting bacterial replication and sustaining regular growth. However, under environmental stress, stressors stimulate bacteria to produce elevated levels of nucleotide-derived molecules, which bind to and significantly activate the ssRNA ribonuclease activity of Csm6. Such activation triggers degradation of mRNA associated with ribosomal protein genes and mycolic acid biosynthesis genes, thereby impairing protein translation and compromising membrane permeability (Fig. 7). Collectively, this cascade disrupts bacterial metabolic homeostasis, thereby increasing cellular vulnerability to antibiotic-mediated stress.

In conclusion, this study underscores the biological function of Csm6 both *in vitro* and *in vivo*. The biochemical activity of Csm6 enables the bacterium to degrade RNA in response to environmental cues, while the CARF domain fine-tunes this response, influencing drug susceptibility. Furthermore, the activity of Csm6 exhibits preference in RNA cleavage. These findings provide critical insights into the broader role of the CRISPR-Cas system in bacterial defense beyond immunity and highlight Csm6’s potential impact on drug resistance mechanisms in *Mtb*.

## Supplementary Material

gkaf622_Supplemental_Files

## Data Availability

Raw RNA sequencing data has been deposited in the Sequence Read Archive (SRA), and are publicly accessible under the accession number PRJNA1218030 (https://www.ncbi.nlm.nih.gov/bioproject/PRJNA1218030). The data that support the findings of this study are available in the methods and/or supplementary material of this article. Materials are available on request from the corresponding author.
