# Supplementary material for: CARF-dependent preferential RNA cleavage by Csm6 increases drug susceptibility of mycobacteria
Source: Nucleic Acids Res. 2025 Jul 2;53(12):gkaf622. doi: 10.1093/nar/gkaf622 (PMC12214007; doi:10.1093/nar/gkaf622)
Supplement: gkaf622_Supplemental_Files [file gkaf622_supplemental_files.zip › 20250606_supplementary_materials.pdf]

CARF-dependent preferential RNA cleavage by Csm6 increases drug susceptibility of mycobacteria

Wenping Wei[1], Chun-Hui Gao[2], Xiaofang Jiang[3], Junjie Qiao[3], Li Zhang[3], Yunjun Yan[1], Guowei Zhao[1], Kaixin Yang[1], Jinyong Yan[1], Min Yang[1]*

[1]Key Laboratory of Molecular Biophysics of the Ministry of Education, College of Life Science and Technology, Huazhong University of Science and Technology, Wuhan, China.

[2]National Key Laboratory of Agricultural Microbiology, College of Resources and Environment, Huazhong Agricultural University, Wuhan, China.

[3] National Key Laboratory of Agricultural Microbiology, College of Life Science and Technology, Huazhong Agricultural University, Wuhan, China.

*To whom correspondence should be addressed:
Min Yang, E-mail: ymyangmin@hust.edu.cn

Supplementary Materials

Table S1 Strains and plasmids used in this study
Table S2 Primers and Oligonucleotides used in this study
Table S3 Primers for real-time PCR (qRT-PCR)
Table S4 Differentially expressed genes in *csm6*-expressing and d*csm6*^{CARF}-expressing strain
Figure S1. The dimeric structure of MtbCsm6 bound to cA6 predicted by AlphaFold
Figure S2. Multiple sequence alignment of Csm6 proteins
Figure S3. KEGG differential expression pathway analysis of *csm6*- and d*csm6*C^{ARF}- expressing strains
Figure S4. Comparison of ribosomal protein gene expression profiles between wild-type, *csm6*-expressing and d*csm6*^{CARF}-expressing strains
Figure S5. Csm6 suppresses the transcription of ribosomal protein genes and mycolic acid synthesis genes
Figure S6. Growth profiles of *csm6*-expressing strain under nutrient limitation and sub-inhibitory antibiotic concentrations
Figure S7. Csm6 expression does not induce SOS response genes
Figure S8. Csm6-mediated drug susceptibility and transcriptional changes in *M. bovis* BCG strains
Figure S9. Metabolomic analysis of nucleotide-derived signaling molecules

Materials and Methods

Protein structural analysis of Csm6

To predict the three-dimensional structure of Csm6 and analyze its interaction with cA6, AlphaFold3 (DeepMind) was used for structural modelling (1). The amino acid sequence of Csm6 was obtained from the corresponding gene sequence and submitted to AlphaFold3 to predict the protein's native conformation. The prediction was carried out using default settings on the AlphaFold3 platform, and the structure was validated based on the predicted confidence scores (pLDDT).

For the analysis of the Csm6-cA6 complex, the predicted Csm6 structure was used as the starting model. The cA6 molecule was docked onto the Csm6 structure using flexible molecular docking tools available in AutoDock Vina 1.5.6 (2). The docking procedure included energy minimization steps to refine the binding conformation and obtain the most probable complex structure. The binding affinity and interaction details between Csm6 and cA6 were assessed using binding energy calculations and visualized with PyMOL 3.1 (3). Key residues involved in the binding interface were identified and analyzed for their role in complex formation.

Drug susceptibility analysis under nutrient-limiting conditions

Cultures of the *csm6*-expressing strain and the pMV261 control strain were grown to mid-log phase in 7H9 medium. Cells were diluted to an initial OD_{600} = 0.05 in fresh 7H9 medium containing 0.0125% glycerol (nutrient-limiting conditions) and treated with sub-inhibitory concentrations of isoniazid (INH: 2.5 µg/mL) or ethambutol (EMB: 2.5 µg/mL). Cultures were incubated at 37°C with shaking for specified durations, and bacterial growth was quantified by measuring OD_{600} at designated time points using a spectrophotometer (Tecan, Switzerland).

Metabolomics analysis

The untargeted metabolomics analysis was conducted using the *csm6*-expressing strain, d*csm6*^{CARF}-expressing strain, and the pMV261 control strain (n = 6) (4). Bacterial samples were extracted with a pre-chilled methanol/acetonitrile/water (2:2:1) solution spiked with isotope-labeled internal standards. After vortexing, samples were ultrasonicated (4°C, 5 min), frozen at -80°C for 30 min, and centrifuged (12,000 ×g, 15 min, 4°C). Supernatants were transferred, re-centrifuged, and analyzed via UPLC-Q-TOF-MS (Agilent 1290/6545).

Chromatographic separation was performed on a Waters BEH Amide column (2.1 × 100 mm, 1.7 µm) with mobile phases: (A) 15 mM ammonium acetate + 0.3% $NH_3{\cdot}H_2O$ in water and (B) 15 mM ammonium acetate + 0.3% $NH_3{\cdot}H_2O$ in 90% acetonitrile. The column temperature was maintained at 40°C with a 2 µL injection volume. Mass spectrometry utilized dual ESI ionization modes: positive (nozzle voltage: 500 V) and negative (nozzle voltage: 1000 V), with a gas temperature of 300°C, sheath gas flow of 11 L/min, and fragmentor voltage of 110 V.

Data preprocessing included missing value imputation, log-transformation, and normalization. Multivariate analyses (PCA, hierarchical clustering) and univariate tests (Student's t-test, $p<0.05$) identified differential metabolites. The boxplot (box-and-whisker plot) analysis was performed using R statistical software (version 4.3.2) with base R functions or packages like ggplot2.

.

Table S1 Strains and plasmids used in this study

**Strains and plasmids**	Relevant genotype or features	Source or reference
Strain		
DH5α	Host for plasmid construction	Novagen
BL21	Host for overexpression	Novagen
M. smegmatis mc^2 155	wild type	ATCC
Ms/pMV261	$mc^2$155 with pMV261	This study
Ms/pMV261-*csm6*	$mc^2$155 with pMV261-*csm6*, *csm6*-expressing strain	This study
Ms/pMV261-d*csm6*^{CARF}	$mc^2$155 with pMV261-*csm6*$^{S73A\text{-}G74A\text{-}T75A\text{-}P76A}$, dCsm6^{CARF} variant	This study
Ms/pMV261-*csm6*^{R324A}	$mc^2$155 with pMV261-*csm6*^{R324A}, dCsm6^{HEPN} variant	This study
Ms/pMV261-*csm6*^{N325A}	$mc^2$155 with pMV261-*csm6*^{N325A}, dCsm6^{HEPN} variant	This study
Ms/pMV261-*csm6*^{H329A}	$mc^2$155 with pMV261-*csm6*^{H329A}, dCsm6^{HEPN} variant	This study
Ms/pMV261-*csm5*	$mc^2$155 with pMV261-*csm5*, *csm5*-expressing strain	This study
Ms/pMV261-*csm4*	$mc^2$155 with pMV261-*csm4*, *csm4*-expressing strain	This study
Ms/pMV261-*csm3*	$mc^2$155 with pMV261-*csm3*, *csm3*-expressing strain	This study
Ms/pMV261-*csm2*	$mc^2$155 with pMV261-*csm2*, *csm2*-expressing strain	This study
Ms/pMV261-*csm1*	$mc^2$155 with pMV261-*csm1*, *csm1*-expressing strain	This study
Ms/pMV261-*cas6*	$mc^2$155 with pMV261-*cas6*, *cas6*-expressing strain	This study
Ms/pMV261-*cas1*	$mc^2$155 with pMV261-*cas1*, *cas1*-expressing strain	This study
Ms/pMV261-*cas2*	$mc^2$155 with pMV261-*cas2*, *cas2*-expressing strain	This study
M. bovis BCG	wild type	ATCC
BCG/pMV261-*csm6*	BCG with pMV261-*csm6*, *csm6*-expressing strain	This study
BCG/pMV261-d*csm6*^{CARF}	BCG with pMV261-*csm6*$^{S73A\text{-}G74A\text{-}T75A\text{-}P76A}$, dCsm6^{CARF} variant	This study
Plasmid		This study
pET28a-SUMO	Kan^r, *lacZ* operon, T7 promotor, N-termial His-Tag, Solubility-enhancing Tag SUMO	Novagen
pETSUMO-*csm6*	*csm6* in *BamH*I-*Hind*III of pET28a-SUMO	This study
pETSUMO-d*csm6*^{CARF}	Csm6 (S73A-G74A-T75A-P76A) mutant	This study
pETSUMO-*csm6*^{H329A}	Csm6 H329A mutant	This study
pMV261	Kan^r, pAL5000 replicon	Novagen
pMV261-*csm6*	*csm6* in EcoRI-XbaI of pMV261	This study
pMV261- d*csm6*^{CARF}	Csm6 S73A-G74A-T75A-P76A	This study
pMV261-*csm6*^{H329A}	*csm6* H329A	This study
pMV261-*csm6*^{R324A}	*csm6* R324A	This study
pMV261-*csm6*^{N325A}	*csm6* N325A	This study

Table S2 Primers and Oligonucleotides used in this study

Name	Sequence (5'-3')	Enzyme	Note
pETCsm6_F	ATAT**GGATCC**GTGCTATTCCTCAGCGCCGA	BamHI	Primer, clone to pET28a-SUMO
pETCsm6_R	AGTC**AAGCTT**TTAGCCCAGCGGTGCCATAT	HindIII	Primer, clone to pET28a-SUMO
pMVCsm6_F	ATAT**GCGGGCCGC**GTGCTATTCCTCAGCGCCGA	NotI	Primer, clone to pMV261
pMVCsm6_R	AGTC**TCTAGA**TTAGCCCAGCGGTGCCATAT	XbaI	Primer, clone to pMV261
dCsm6^{CARF} _F	CTGCTGAATACCAGTGCCGCCGCCGCTGCGATGCAGGCGGCGCTGGT		Mutagenesis
dCsm6^{CARF} _R	CGCCGCCTGCATCGCAGCGGCGGCGGCACTGGTATTCAGCAGAATGG		Mutagenesis
Csm6_R324A_F	TTCGAGAGCCGGGTCGCCAACACCGCAGCACACGAG		Mutagenesis
Csm6_R324A_R	GTGTGCTGCGGTGTTGGCGACCCGGCTCTCGAACCT		Mutagenesis
Csm6_N325A_F	GAGAGCCGGGTCCGCGCCACCGCAGCACACGAGATC		Mutagenesis
Csm6_N325A_R	CTCGTGTGCTGCGGTGGCGCGGACCCGGCTCTCGAA		Mutagenesis
Csm6_H329A_F	CGCAACACCGCAGCAGCCGAGATCGTCTCAATCAGT		Mutagenesis
Csm6_H329A_R	GATTGAGACGATCTCGGCTGCTGCGGTGTTGCGGAC		Mutagenesis
pMVCsm5_F	ATAT**GCGGGCCGC**ATGAACACCTACCTGAAGCC	NotI	Primer, clone to pMV261
pMVCsm5_R	AGTC**TCTAGA**TCATTCGGCTCTCCTGATCG	XbaI	Primer, clone to pMV261
pMVCsm4_F	ATAT**GCGGGCCGC**ATGAACTCGCGGCTGTTTAG	NotI	Primer, clone to pMV261
pMVCsm4_R	AGTC**TCTAGA**TCATGCGGCGGACTCCGGGA	XbaI	Primer, clone to pMV261
pMVCsm3_F	ATAT**GCGGGCCGC**ATGACTACGAGCTACGCCAA	NotI	Primer, clone to pMV261
pMVCsm3_R	AGTC**TCTAGA**CTAAACAGCCGCGAGTTCAT	XbaI	Primer, clone to pMV261
pMVCsm2_F	ATAT**GCGGGCCGC**ATGAGCGTCATCCAAGACGA	NotI	Primer, clone to pMV261
pMVCsm2_R	AGTC**TCTAGA**TCACTTGTCCTTCGGATCGA	XbaI	Primer, clone to pMV261
pMVCsm1_F	ATAT**GCGGGCCGC**ATGAACCCGCAACTCATCGA	NotI	Primer, clone to pMV261
pMVCsm1_R	AGTC**TCTAGA**TCATTCGGACTCCTCCTTGC	XbaI	Primer, clone to pMV261
pMVCas6_F	ATAT**GCGGGCCGC**TTGGCTGCTCGCCGAGGCGG	NotI	Primer, clone to pMV261
pMVCas6_R	AGTC**AAGCTT**TCATGGCTTCGGTACGCATT	HindIII	Primer, clone to pMV261
pMVCas1_F	ATAT**GCGGGCCGC**ATGGTGCAGCTGTATGTCTC	NotI	Primer, clone to pMV261
pMVCas1_R	AGTC**TCTAGA**TTAGGCTCCGGATGGCTCGG	XbaI	Primer, clone to pMV261

pMVCas2_F	ATAT**<u>GCGGGCCGC</u>**ATGCCCACTCGCAGCCGTGA	NotI	Primer, clone to pMV261
pMVCas2_R	AGTC**<u>TCTAGA</u>**TCAAAAGAACACAAACTCCT	XbaI	Primer, clone to pMV261
RNA1	ACUGCAACGCAAUAUACCAUAGCU		3’-Cy5 modified RNA
RNA2	AGCUAUGGUAUAUUGCGUUGCAGU		RNA
DNA1	ACTGCAACGCAATATACCATAGCT		3’-Cy5 modified DNA
DNA2	AGCTATGGTATATTGCGTTGCAGT		DNA

Table S3 Primers for real-time PCR(RT-PCR)

Name	Sequence (5'-3')	Note
Csm6_RT_F	TCAAGTGAGCAATCTGATCCG	
Csm6_RT_R	ATGTACTCAGCGACCTTGTTC	
MSMEG_1339-RTF	AAGCACCGGAACTACATCAC	These primers are used in RT-PCR to amplify genes that regulate ribosome pathway in *M. smegmatis* strain. Specifically, *sigA* gene was employed as internal reference of PCR experiments.
MSMEG_1339-RTR	ACTCTTTGTGCGGCTGAT	
MSMEG_1435-RTF	CGACCATGAGGCCATTGAT	
MSMEG_1435-RTR	GACGCAGTACACGTTCTTCT	
MSMEG_1436-RTF	AGAACCTGAAGGTGCACAAG	
MSMEG_1436-RTR	TTCTCGCCTCGCTTGATTG	
MSMEG_1465-RTF	ACGGCAGCTACATCAAGTTC	
MSMEG_1465-RTR	ACTTCTTCTCGCGCAGTTC	
MSMEG_4571-RTF	CGTAACCAGTCGGTGAAGTC	
MSMEG_4571-RTR	TAGCAACTCGGATGCCTTG	
MSMEG_5489-RTF	GCCCAAGCGCAGAATGT	
MSMEG_5489-RTR	TCGACCAGACCCAGACG	
SigA_RTF	GGAGAAGTTCGACTACACC	
SigA_RTR	GTTGATCACCTCGACCATGT	
MSMEG_3150-RTF	CCTGTTCGGTGTTCAATGTG	These primers are used in RT-PCR to amplify genes that regulate mycolic acid biosynthesis pathway in *M. smegmatis* strain.
MSMEG_3150-RTR	GGAATGCGTCCTTGGAGATG	
MSMEG_3151-RTF	TTCTGCCGATCATGAATCCG	
MSMEG_3151-RTR	CGACGGTCATCCAGTTGTAG	
MSMEG_1350-RTF	TTTTACGAGCTGTTCCTGGG	
MSMEG_1350-RTR	TGTCGAGCAGTGTCATGC	
MSMEG_1340-RTF	ATCTGCATCTTCGGCTATCAG	
MSMEG_1340-RTR	TCGCAGTACAAACGGTCAC	
MSMEG_1932-RTF	TTATTCGCCGTATGACACCG	
MSMEG_1932-RTR	TCGTAGTCCTGATAGTCACCG	
MSMEG_6398-RTF	AGTTCATCTACGCGGCATC	
MSMEG_6398-RTR	TTGTTCGGATCGTTGGTCG	
MSMEG_6399-RTF	GTTCTACAACTCGGGCATCTC	
MSMEG_6399-RTR	GAACGTCTCCCACTTATAGGTG	
MSMEG_2078-RTF	CATCTGGGTGTACTGCGG	
MSMEG_2078-RTR	ATGTAGTTGTCGCGGAAGG	
MSMEG_6393-RTF	TTCCAGAACATCCTCAAGTCG	
MSMEG_6393-RTR	GATGACGAGGTCCTTGACG	
MSMEG_6392-RTF	GCTACCTCACCGACATCAAG	
MSMEG_6392-RTR	CTCCAACGCTTCCCACG	
MSMEG_4757-RTF	GTACCGAGCAGACCGAAAG	
MSMEG_4757-RTR	CAGCACCTTCATCTCCATCTC	
BCGsigA-RTF	CGGTGATTTCGTCTGGGATG	These primers are used in RT-PCR to amplify genes that regulate mycolic acid biosynthesis pathway in *M. bovis* BCG strain. Specifically, *sigA* gene was employed as internal reference of PCR experiments.
BCGsigA-RTR	TTGCCGATCTGTTTGAGGTAG	
inhA-RTF	ATCCACATCTCGGCGTATTC	
inhA-RTR	CGACCGTCATCCAGTTGTAG	
hadA-RTF	GAACTCGGGTATAAGGGCTTG	
hadA-RTR	CGGTTTCTCGAATTTCAGCAC	

fbpD-RTF	CGAGGCGATGGGTAACAG	
fbpD-RTR	AGTCGAAGTGTCCGTTGTG	
fas-RTF	TGTACCACGGCAATCTGTTG	
fas-RTR	GGCAATGATATTCGGCAAGAC	
pks13-RTF	CATTCCGTCTACTTCACCCAC	
pks13-RTR	GGATCAACTGGGCGTCATG	
fadD32-RTF	AAAGATCAGGTTCCCAGCC	
fadD32-RTR	AAGTCAGACCACAAGATGTCG	
cmrA-RTF	AGAGATTCGCTGAAGGTGTTC	
cmrA-RTR	GGCATCTTGATTTCTGGTTCG	
fbpC-RTF	CTGTCCTGGTTTACGGTCTG	
fbpC-RTR	CCTGGAACTGGACCTTGATG	
fabG1-RTF	CATTCGTATCCCGTTCAGTCC	
fabG1-RTR	CACATTCGACGCCAAACAG	

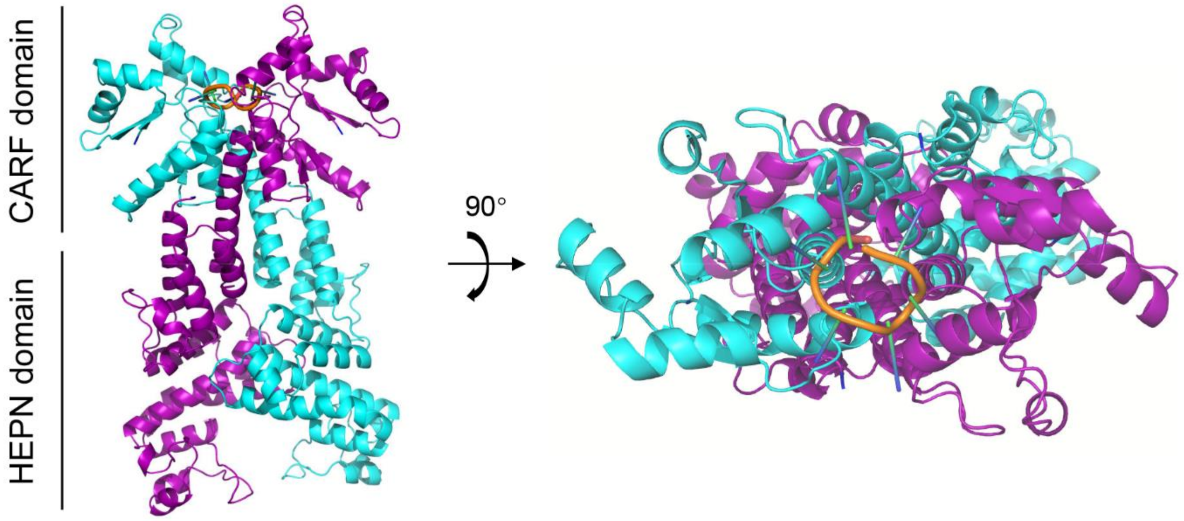

Supplementary Figure 1. The dimeric structure of MtbCsm6 bound to cA6 predicted by AlphaFold. In the left-side view, the protein structure is depicted with coiled, ribbon-like structures in purple and cyan-green, representing different domains. The upper section is labeled "CARF domain," and the lower section is labeled "HEPN domain," clearly illustrating the relative positions and shapes of the two domains. A black rotational arrow in the middle, labeled "90°," indicates the rotation angle from the left-side view to the right-side view. The right-side view shows the structure after a 90° rotation, with the protein structure remaining composed of purple and cyan-green coiled ribbons. An orange molecule, cA6, is located at the center of the CARF domain.

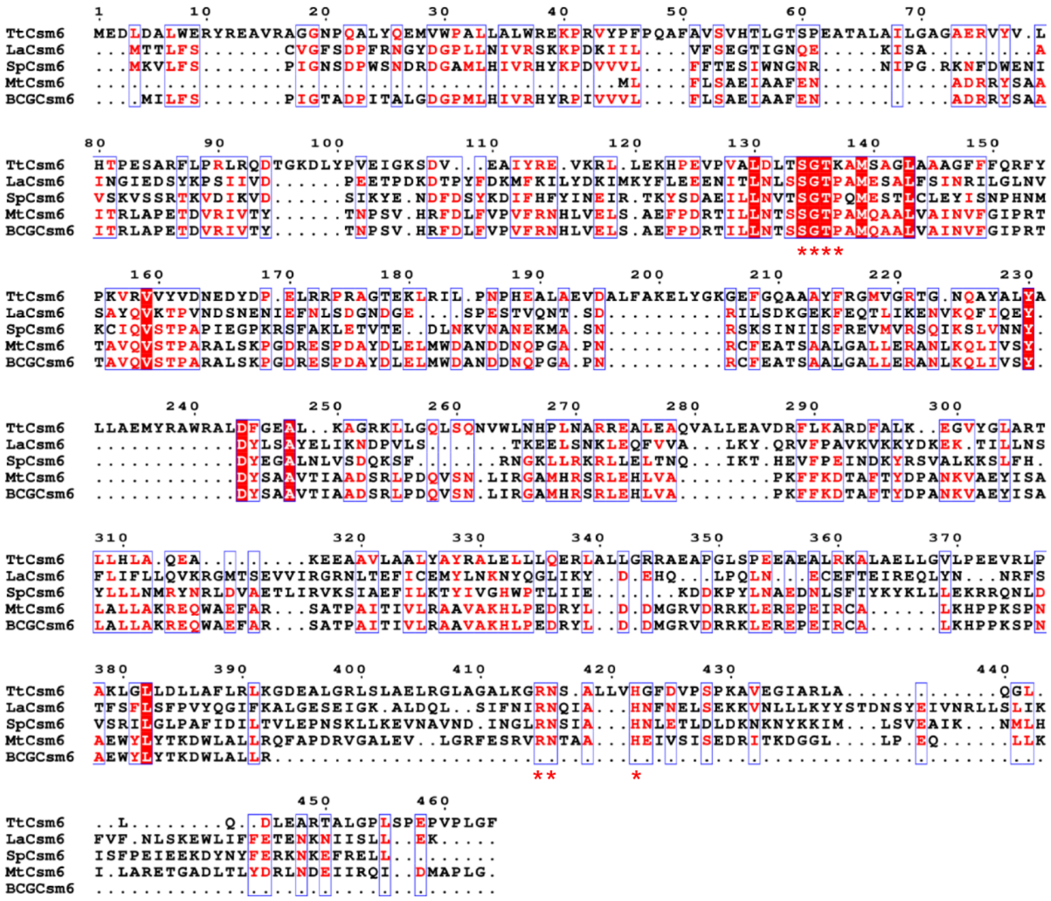

Supplementary Figure 2. Multiple sequence alignment of Csm6 proteins. The proteins analyzed were from *Thermus thermoophilus* (TtCsm6), *Staphylococcus pseudintermedius* (SpCsm6), *Lactobacillus acidipiscis* (LaCsm6), *Mycobacterium tuberculosis* (MtCsm6), and *M. bovis* BCG (BCGCsm6). Amino acid residues at the active sites of the CARF and HEPN domains are marked with asterisks. The SGTP motif in the CARF domain and the R-$X_{4\text{-}6}$-H motif in the HEPN domain are highlighted with red asterisks.

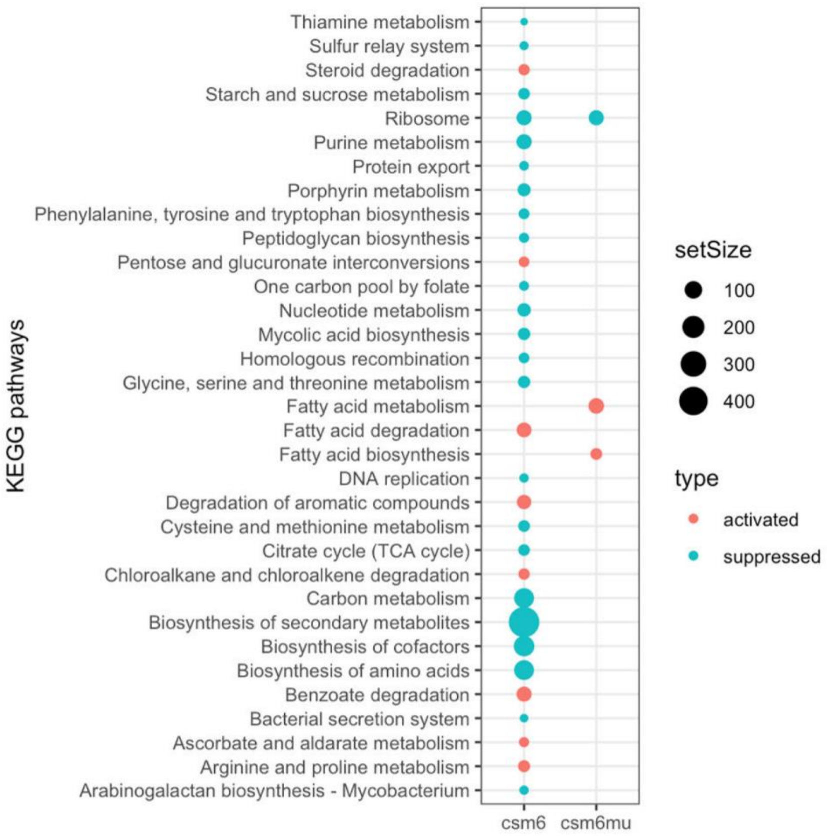

Supplementary Figure 3. KEGG pathway enrichment analysis of differentially expressed metabolic pathways in *csm6*-expressing (csm6), and d*csm6*^{CARF}-expressing (csm6mu) strains, as compared with the pMV261 control strain. Pathways highlighted in Coral Red represent activated pathways, while those in Cyan Blue indicate inhibited pathways. The size of the circles is proportional to the number of enriched genes in each pathway.

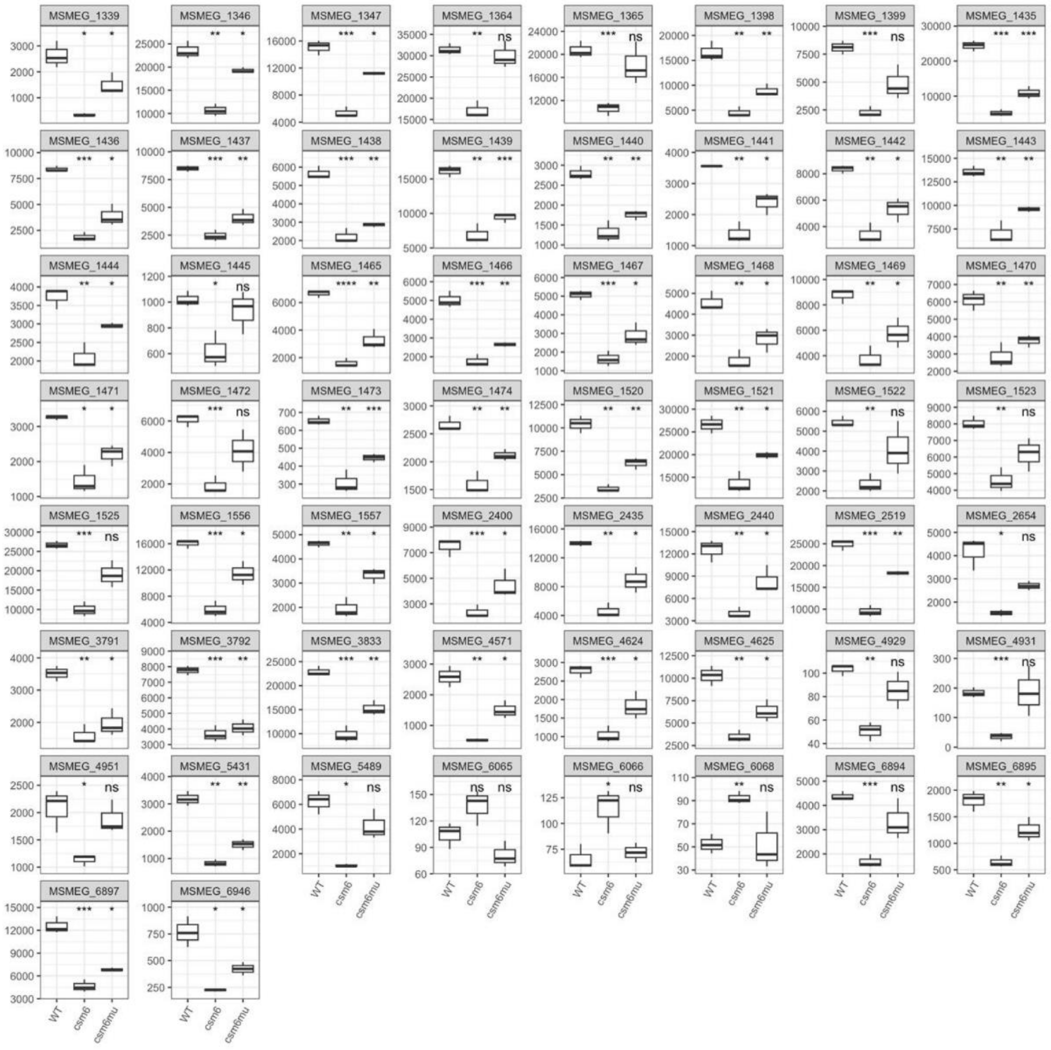

Supplementary Figure 4. Comparison of ribosomal protein gene expression profiles between pMV261 (WT), *csm6*-expressing (csm6), and d*csm6*^{CARF}-expressing (csm6mu) strains. The gene expression levels of ribosomal proteins were measured and compared across the three strains. Statistical significance is indicated by the following annotations: **P* < 0.05, ***P* < 0.01, ****P* < 0.001, highlighting the differences in expression between the strains.

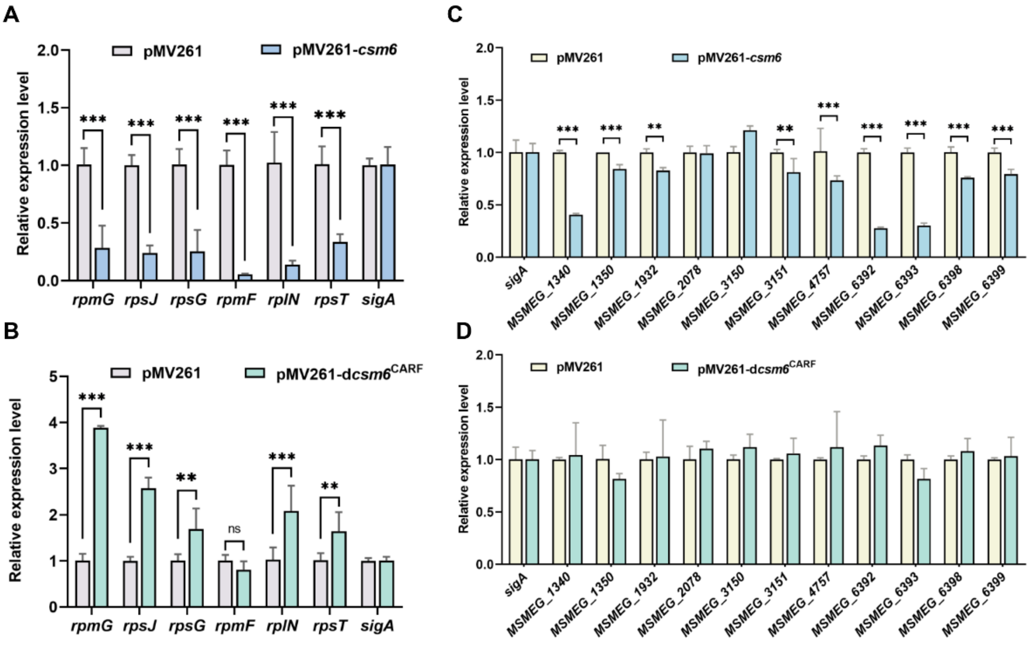

Supplementary Figure 5. Csm6 suppresses the transcription of ribosomal protein genes and mycolic acid synthesis genes. Expression levels of ribosomal protein genes in *csm6*-expressing strain (A) and d*csm6*^{CARF}-expressing strain (B). Relative expression levels of *rplC*, *rplN*, *rpmF*, *rpmG*, *rpsJ* and *rpsT* genes were normalized to *sigA*. (B) Expression levels of mycolic acid biosynthesis genes in *csm6*-expressing strain (C) and d*csm6*^{CARF}-expressing strain (D). Relative mRNA levels of mycolic acid biosynthesis-related genes in empty vector control strains (pMV261; light blue) and *csm6*-expressing strains (pMV261-csm6; dark blue), normalized to *sigA*. Data are mean ± SD. *P*-values were calculated by GraphPad Prism 8 with independent samples two-tailed Student's *t*-test. The asterisks represent significant differences between the two groups (**P* < 0.05, ***P* < 0.01, ****P* < 0.001).

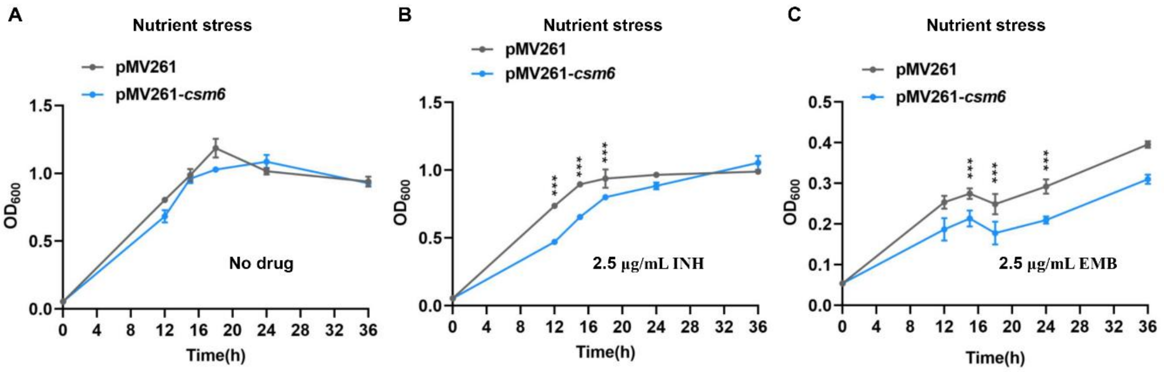

Supplementary Figure 6. Growth profiles of *csm6*-expressing strain under nutrient limitation and sub-inhibitory antibiotic concentrations. Growth profiles of the *csm6*-expressing strain and control strain were compared in a nutrient-limited medium (0.25% glycerol): (A) no drug; (B) 2.5 μg/mL INH; and (C) 2.5 μg/mL EMB. Data are mean ± SD. *P*-values were calculated by GraphPad Prism 8 with independent samples two-tailed Student's *t*-test. The asterisks represent significant differences between the two groups (*$P < 0.05$, **$P < 0.01$, ***$P < 0.001$).

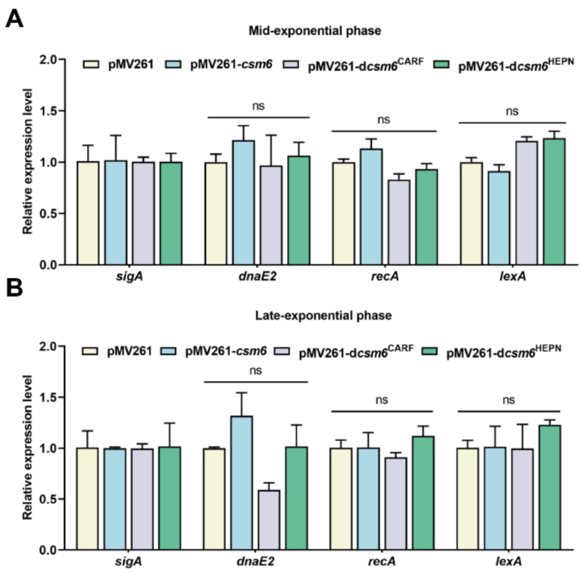

Supplementary Figure 7. Csm6 expression does not induce SOS response genes. Relative expression of SOS response markers (*dnaE2*, *recA*, *lexA*) measured by qPCR at mid-exponential phase **(A)** and late-exponential phase **(B)**. Expression analysis of the SOS response genes in the *csm6*-expressing strain (pMV261-*csm6*), the d*csm6*^{CARF}-expressing strain (pMV261-d*csm6*^{CARF}), the d*csm6*^{HEPN}-expressing strain (pMV261-d*csm6*^{HEPN}), and the pMV261 control strain. Error bars represent SD from three biological replicates. *P*-values were calculated by GraphPad Prism 8 with independent samples two-tailed Student's *t*-test. the difference between treated and control groups was marked as *ns* ($P > 0.05$).

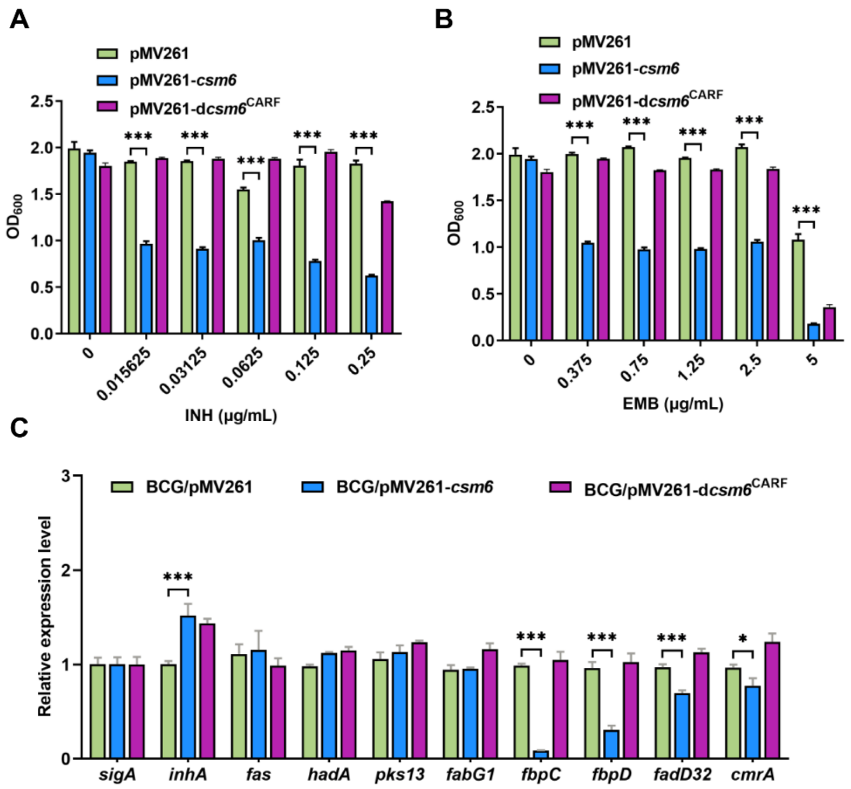

Supplementary Figure 8. Csm6-mediated drug susceptibility and transcriptional changes in *M. bovis* BCG strains. Drug susceptibility assays to INH (A) and EMB (B). *M. bovis* BCG strains carrying pMV261 vector (green), wild-type *csm6* (blue), or CARF-domain mutant *csm6* (purple) were grown in 7H9 medium with graded drug concentrations (INH: 0-2.5 μg/mL; EMB: 0-5 μg/mL) at 37°C for 5 days, followed by OD_{600} measurement. (C) qRT-PCR analysis of mycolic acid biosynthesis genes. Relative mRNA levels of target genes in pMV261 control strain (green), *csm6*-expressing strain (blue), and d*csm6*^{CARF}-expressing strain (purple) normalized to *sigA*. *P*-values were calculated by GraphPad Prism 8 with independent samples two-tailed Student's *t*-test. The asterisks represent significant differences between the two groups (*$P < 0.05$, **$P < 0.01$, ***$P < 0.001$).

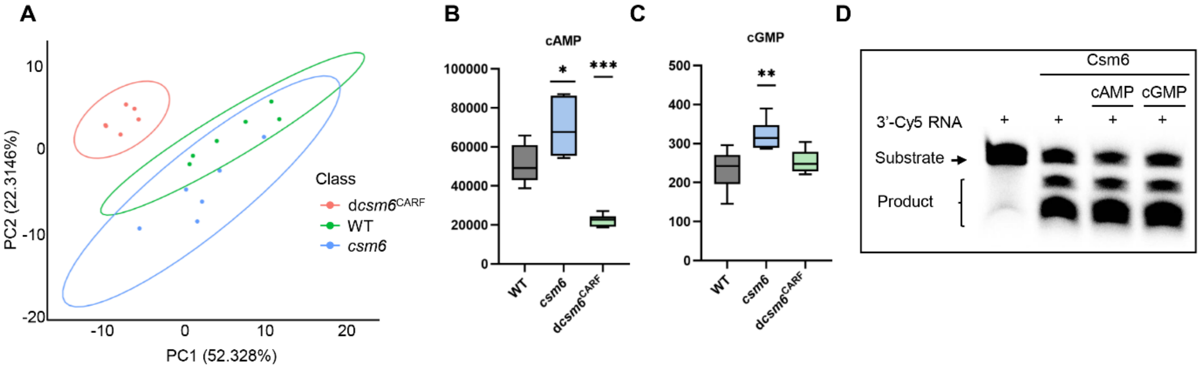

Supplementary Figure 9. Metabolomic analysis of nucleotide-derived signaling molecules. (A) Principal component analysis (PCA) of targeted metabolomics in empty vector control strain, *csm6*-expressing strain, and d*csm6*^{CARF}-expressing strain**.** The circles represent the 95% confidence interval. (B-C) Targeted quantification of intracellular cAMP (B) and cGMP (C) levels. (*P < 0.05, **P < 0.01, ***P < 0.001; two-tailed Student's t-test). (D) *In vitro* RNase activity assay of purified Csm6. No cleavage enhancement was observed with 1 mM cAMP or cGMP supplementation using a 3′-Cy5-labeled RNA substrate. Reactions were incubated at 37°C for 30 min and analyzed by denaturing PAGE. Data are representative of three replicates.

Reference

1. Abramson, J., Adler, J., Dunger, J., Evans, R., Green, T., Pritzel, A., Ronneberger, O., Willmore, L., Ballard, A. J., Bambrick, J., Bodenstein, S. W., Evans, D. A., Hung, C. C., O'Neill, M., Reiman, D., Tunyasuvunakool, K., Wu, Z., Žemgulytė, A., Arvaniti, E., Beattie, C., Bertolli, O., Bridgland, A., Cherepanov, A., Congreve, M., Cowen-Rivers, A. I., Cowie, A., Figurnov, M., Fuchs, F. B., Gladman, H., Jain, R., Khan, Y. A., Low, C. M. R., Perlin, K., Potapenko, A., Savy, P., Singh, S., Stecula, A., Thillaisundaram, A., Tong, C., Yakneen, S., Zhong, E. D., Zielinski, M., Žídek, A., Bapst, V., Kohli, P., Jaderberg, M., Hassabis, D., and Jumper, J. M. (2024) Accurate structure prediction of biomolecular interactions with AlphaFold 3. *Nature* **630**, 493-500
2. Trott, O., and Olson, A. J. (2010) AutoDock Vina: improving the speed and accuracy of docking with a new scoring function, efficient optimization, and multithreading. *J. Comput. Chem.* **31**, 455-461
3. Branden, C., and Tooze, J. (1991) Introduction to protein structure.
4. Li, Z., Zhang, H. X., Li, Y., Lam, C. W. K., Wang, C. Y., Zhang, W. J., Wong, V. K. W., Pang, S. S., Yao, M. C., and Zhang, W. (2019) Method for Quantification of Ribonucleotides and Deoxyribonucleotides in Human Cells Using (Trimethylsilyl)diazomethane Derivatization Followed by Liquid Chromatography-Tandem Mass Spectrometry. *Anal. Chem.* **91**, 1019-1026
